# Genetic modulation of Valencia sweet orange field performance by 50 rootstocks under huanglongbing-endemic conditions

**DOI:** 10.3389/fpls.2023.1061663

**Published:** 2023-02-08

**Authors:** Kim D. Bowman, Greg McCollum, Danelle K. Seymour

**Affiliations:** ^1^ U.S. Horticultural Research Laboratory, Agricultural Research Service, United States Department of Agriculture, Ft. Pierce, FL, United States; ^2^ Department of Botany and Plant Sciences, University of California, Riverside, Riverside, CA, United States

**Keywords:** citrus, breeding, field testing, fruit quality, huanglongbing, heritability

## Abstract

Although the citrus scion cultivar primarily determines the characteristics of the fruit, the rootstock cultivar of the graft combination has a major role in determining the horticultural performance of the tree. The disease huanglongbing (HLB) is particularly devastating to citrus, and the rootstock has been demonstrated to modulate tree tolerance. However, no existing rootstock is entirely suitable in the HLB-endemic environment, and citrus rootstocks are particularly challenging to breed because of a long life cycle and several biological characteristics that interfere with breeding and commercial use. This study with Valencia sweet orange scion documents the multi-season performance of 50 new hybrid rootstocks and commercial standards in one trial that forms the first wave of a new breeding strategy, with the aim of identifying superior rootstocks for commercial use now, and mapping important traits to be used in selection for the next generation of outstanding rootstocks. A large assortment of traits were quantified for all trees in the study, including traits associated with tree size, health, cropping, and fruit quality. Among the quantitative traits compared between rootstock clones, all except one were observed to have significant rootstock influence. Multiple progeny from eight different parental combinations were included in the trial study, and significant differences between parental combinations of the rootstocks were observed for 27 of the 32 traits compared. Pedigree information was integrated with quantitative trait measurements to dissect the genetic components of rootstock-mediated tree performance. Results suggest there is a significant genetic component underlying rootstock-mediated tolerance to HLB and other critical traits, and that integration of pedigree-based genetic information with quantitative phenotypic data from trials should enable marker-based breeding approaches for the rapid selection of next-generation rootstocks with superior combinations of traits that are needed for commercial success. The current generation of new rootstocks included in this trial is a step toward this goal. Based on results from this trial, the new hybrids US-1649, US-1688, US-1709, and US-2338 were considered the four most promising new rootstocks. Release of these rootstocks for commercial use is being considered, pending the evaluation of continuing performance in this trial and the results from other trials.

## Introduction

Worldwide production of citrus crops is primarily by a fruiting scion cultivar grafted onto a separate cultivar selected for favorable rootstock traits. In addition to providing for rapid tree propagation and tree size control, the specialized rootstock variety allows selection for a strong root system, high fruit production, and tolerance to a wide range of biotic and abiotic problems independent of the fruiting variety. Many common rootstock clones, both of natural origin and the product of breeding programs, are relatively widespread across citrus production regions, while others are very localized in commercial use (Bowman and Joubert, 2020). As a result of the strong rootstock effects, citrus growers in most production regions regard identification of superior new rootstocks to be an important need.

Citrus production is negatively affected by numerous pests and diseases associated with the root system. Generally, the disease huanglongbing (HLB; also known as citrus greening) associated with the bacteria *Candidatus* Liberibacter asiaticus (CLas) is considered the most damaging problem anyplace where it is found, or the greatest threat to areas where it has not yet spread. Most major commercial citrus scion cultivars are heavily damaged by HLB (Bove, 2006), but some rootstocks are known to have tolerance to the disease (Albrecht and Bowman, 2011; Albrecht and Bowman, 2012; Bowman and Albrecht, 2020). For trees with HLB-sensitive scions in an HLB-endemic environment, use of the tolerant rootstocks can significantly improve overall tree health, and increase fruit production and quality (Bowman and McCollum, 2015; Bowman et al., 2016a; Bowman et al., 2016b). The development and use of more HLB-tolerant rootstocks appears a reasonable strategy to improve fruit quality and productivity in infected regions.

Work has been conducted over many years at several institutions to develop and evaluate superior new citrus rootstock cultivars, including at the United States Department of Agriculture (USDA) (Wutscher and Bowman, 1999; Bowman and Rouse, 2006; Bowman et al., 2016b, Bowman et al., 2021), University of Florida (Castle, 2010; Grosser et al., 2016; Grosser et al., 2020; Kunwar et al., 2021), University of California (Bitters, 1986; Roose et al., 1989), Valencian Institute of Agricultural Research (IVIA) in Spain (Forner-Giner et al., 2003; Forner-Giner et al., 2014; Martínez-Cuenca et al., 2016), CRA-Research Center for Citriculture and Mediterranean Crops (CRA-ACM; CREA) in Italy (Russo and Reforgiato Recupero, 1984; Reforgiato Recupero et al., 2009), and in Brazil under the Brazilian Agricultural Research Corporation (EMBRAPA), The Sylvio Moreira Citrus Culture Center, Fundecitrus-IDR-Paraná, and University of São Paulo (Cantuarias-Avilés et al., 2010; Costa et al., 2020a; Costa et al., 2020b; Soratto et al., 2020; Domingues et al., 2021). These efforts have involved a combination of testing rootstock clones already in existence (including those imported from other regions) and creating new clones by sexual hybridization between two parental species. Over the last 35 years, several programs also used somatic hybridization to create new rootstock clones included in evaluations (Grosser et al., 1998; Grosser et al., 2004; Dambier et al., 2011; Grosser and Gmitter, 2011).

Although citrus breeding has been a focus of international research for over 100 years, the long lifecycle and widespread apomixis in most citrus species has limited development of foundational genetic information associated with rootstocks and effective tools for genetic evaluation of a broad array of citrus parents and progeny (Bowman et al., 2021). Recent work has made important strides to bring molecular genetic tools to bear in the genetic improvement of citrus (Gois et al., 2016; Imai et al., 2016; Minamikawa et al., 2017; Lima et al., 2018; Imai et al., 2019; Raveh et al., 2020; Shimizu et al., 2020), but efforts to date have generally focused on scion germplasm and traits most relevant to genetic improvement of citrus scions rather than rootstocks. The nearly-universal use of citrus rootstocks, the observed importance of the rootstock cultivar in citrus trees, and the broad array of root-associated threats to citrus production all provide strong motivation for a more systematic strategy for citrus rootstock breeding, along with the development and utilization of molecular tools to improve efficiency and success.

This study is one component of the new USDA SuperSour strategy (Bowman et al., 2021) being implemented to accelerate the development of superior new HLB-tolerant rootstocks, and provide better molecular tools for the next generation of citrus rootstock breeding. This trial is the first of a series of rootstock trials designed to: 1) identify superior new hybrid rootstocks for the HLB-endemic environment, 2) identify the attributes of different parental combinations to help direct future rootstock crosses, and 3) develop molecular genetic tools that can be used to pre-select the most promising next-generation hybrid rootstocks. Use of these tools will allow long-term rootstock field trials to be focused on hybrids with the best genetic traits and highest potential to be outstanding new rootstocks for commercial use. This trial is a replicated field evaluation of 46 new hybrid rootstocks, including a preliminary assessment of field performance, and determination of important factors in the rootstock breeding program such as heritability of traits, breeding values among the parents and progeny, and assessing the suitability of each parental combination to provide the desired rootstock traits.

## Materials and methods

### Plant materials

New hybrid rootstocks were created within the USDA-ARS citrus rootstock breeding program through sexual hybridization among parental material at the USDA Whitmore Foundation Farm (Groveland, Florida) and the USDA Picos Farm (Ft. Pierce, Florida). Forty-six new hybrids were selected for use in the study (**Table 1**). A standardized abbreviated parentage is indicated for each hybrid, which will be used in the results and discussion sections of the manuscript. In addition to the new hybrids, four commercially available rootstocks were included as standards in this trial (as well as the other trials under the SuperSour strategy): Standard sour orange, Swingle citrumelo, Cleopatra mandarin, and Ridge sweet orange. While the latter two rootstocks are of relatively minor commercial importance in Florida, the rootstocks Standard sour orange and Swingle citrumelo have been widely used for many years, and remain two of the most common rootstocks for new propagations (Rosson, 2020).

**Table 1:** Rootstocks tested.

Rootstock	Parentage^a^	Short Parentage
Cleopatra	*Citrus reticulata*	
Ridge	*Citrus sinensis*	
Sour orange	*Citrus aurantium*	
Swingle	*Citrus paradisi* × *Poncirus trifoliata*	
US-1103	*Microcitrus warburgiana* × *C. aurantium* ‘Chinotto’	Mw × Chinotto
US-1649	*Citrus maxima* ‘Hirado’ × *C. reticulata* ‘Sunki’	Cm × Sunki
US-1653	*C. maxima* ‘Hirado’ × *C. reticulata* ‘Sunki’	Cm × Sunki
US-1672	*C. maxima* ‘Hirado’ × C. *reticulata* ‘Cleopatra’	Cm × Cleopatra
US-1673	*C. maxima* ‘Hirado’ × *Citrus tachibana*	Cm × Tachibana
US-1676	*C. maxima* ‘Hirado’ × *C. tachibana*	Cm × Tachibana
US-1678	*C. maxima* ‘Hirado’ × *C. tachibana*	Cm × Tachibana
US-1679	*C. maxima* ‘Hirado’ × *C. tachibana*	Cm × Tachibana
US-1680	*C. maxima* ‘Hirado’ × *C. tachibana*	Cm × Tachibana
US-1681	*C. maxima* ‘Hirado’ × *C. tachibana*	Cm × Tachibana
US-1687	*C. maxima* ‘Hirado’ × *C. reticulata* ‘Cleopatra’	Cm × Cleopatra
US-1688	*C. maxima* ‘Hirado’ × *C. reticulata* ‘Cleopatra’	Cm × Cleopatra
US-1691	*C. maxima* ‘Hirado’ × *C. reticulata* ‘Cleopatra’	Cm × Cleopatra
US-1694	*C. maxima* ‘Hirado’ × *C. reticulata* ‘Cleopatra’	Cm × Cleopatra
US-1701	*C. maxima* ‘Mato’ × *C. reticulata* ‘Shekwasha’	Cm × Shekwasha
US-1709	*C. maxima* ‘Mato’ × *C. reticulata* ‘Shekwasha’	Cm × Shekwasha
US-1790	*C. reticulata* ‘Ninkat’ × *P. trifoliata* ‘Rich 5-2’	Ninkat × Pt
US-2102	*C. reticulata* ‘Sunki’ × US-802 (*C. maxima* × *P. trifoliata*)	Sunki × (Cm × Pt)
US-2104	*C. reticulata* ‘Sunki’ × US-802 (*C. maxima* × *P. trifoliata*)	Sunki × (Cm × Pt)
US-2106	*Citrus shunkokan* × *C. maxima* ‘Hirado’	Shunkokan × Cm
US-2107	*C. shunkokan* × *C. maxima* ‘Hirado’	Shunkokan × Cm
US-2109	*C. maxima* ‘Hirado’ × US-896 (*C. reticulata* ‘Cleopatra’ × *P. trifoliata*)	Cm × (Cleopatra × Pt)
US-2111	*C. maxima* ‘Hirado’ × US-942 (*C. reticulata* ‘Sunki’ × *P. trifoliata*)	Cm × (Sunki × Pt)
US-2123	*C. maxima* ‘Hirado’ × US-852 (*C. reticulata* ‘Changsha’ × *P. trifoliata*)	Cm × (Changsha × Pt)
US-2132	*C. maxima* ‘Hirado’ × US-942 (*C. reticulata* ‘Sunki’ × *P. trifoliata*)	Cm × (Sunki × Pt)
US-2135	*C. maxima* ‘Hirado’ × US-942 (*C. reticulata* ‘Sunki’ × *P. trifoliata*)	Cm × (Sunki × Pt)
US-2136	*C. maxima* ‘Hirado’ × US-942 (*C. reticulata* ‘Sunki’ × *P. trifoliata*)	Cm × (Sunki × Pt)
US-2137	*C. maxima* ‘Hirado’ × US-942 (*C. reticulata* ‘Sunki’ × *P. trifoliata*)	Cm × (Sunki × Pt)
US-2143	*C. shunkokan* × *C. maxima* ‘Hirado’	Shunkokan × Cm
US-2152	*C. reticulata* ‘Sunki’ × US-802 (*C. maxima* × *P. trifoliata*)	Sunki × (Cm × Pt)
US-2153	*C. reticulata* ‘Sunki’ × US-802 (*C. maxima* × *P. trifoliata*)	Sunki × (Cm × Pt)
US-2156	*C. reticulata* ‘Sunki’ × US-802 (*C. maxima* × *P. trifoliata*)	Sunki × (Cm × Pt)
US-2158	*C. reticulata* ‘Sunki’ × US-802 (*C. maxima* × *P. trifoliata*)	Sunki × (Cm × Pt)
US-2173	*C. reticulata* ‘Sunki’ × US-802 (*C. maxima* × *P. trifoliata*)	Sunki × (Cm × Pt)
US-2214	*C. shunkokan* × *C. maxima* ‘Hirado’	Shunkokan × Cm
US-2229	*C. shunkokan* × *C. maxima* ‘Hirado’	Shunkokan × Cm
US-2234	*C. shunkokan* × *C. maxima* ‘Hirado’	Shunkokan × Cm
US-2240	*C. shunkokan* × *C. maxima* ‘Hirado’	Shunkokan × Cm
US-2250	*C. shunkokan* × *C. maxima* ‘Hirado’	Shunkokan × Cm
US-2257	*C. shunkokan* × *C. maxima* ‘Hirado’	Shunkokan × Cm
US-2272	*C. shunkokan* × *C. maxima* ‘Hirado’	Shunkokan × Cm
US-2280	*C. shunkokan* × *C. maxima* ‘Hirado’	Shunkokan × Cm
US-2293	*C. reticulata* ‘Kunembo’ × *C. maxima* ‘Hirado’	Kunembo × Cm
US-2336	*C. maxima* ‘Hirado’ × US-852 (*C. reticulata* ‘Changsha’ × *P. trifoliata*)	Cm × (Changsha × Pt)
US-2338	*C. maxima* ‘Hirado’ × US-852 (*C. reticulata* ‘Changsha’ × *P. trifoliata*)	Cm × (Changsha × Pt)
US-2343	*C. maxima* ‘Hirado’ × US-852 (*C. reticulata* ‘Changsha’ × *P. trifoliata*)	Cm × (Changsha × Pt)

a Clone identified as C. maxima ‘Hirado’ is a Florida selection from seedlings of ‘Hirado Buntan’. Clone identified as C. maxima ‘Mato’ is a Florida selection from seedlings of ‘Mato Buntan’.

### Propagation of trees

Rootstocks for all trees included in the trial (with one exception, as indicated below) were propagated August-November 2013 by stem cuttings on a mist bench and from greenhouse source material, using racks of 3.8 cm × 21 cm cells (Cone-tainers; Stuewe and Sons, Tangent, OR, USA) and soilless potting mix (Pro Mix BX; Premier Horticulture, Inc., Quakertown, PA, USA), as previously described (Bowman and Albrecht, 2017). For rootstock US-1790, nucellar true-to-type seedlings were used for propagation because cuttings were not available. Healthy liners of the 50 rootstocks were transplanted into 2.54 liter pots (Treepots; Stuewe and Sons) using soilless potting mix (Pro Mix BX). Rootstock liners were budded during spring 2014 in the certified greenhouse nursery at USDA, ARS, Ft. Pierce using certified budwood of the Valencia sweet orange clone 1-14-19, the most widely used Valencia clone in Florida. Common greenhouse methods were used for nursery tree care during propagation and until trees were planted into the field.

### Trial location and design

The rootstock trial was planted into the field at the USDA Picos farm (Ft. Pierce, St Lucie County, Florida) in October 2014 at latitude 27.437062˚, longitude -80.427313˚, and thereafter was identified as “Picos 2014”. Soil in the experimental block is classified as Riviera fine sand. Ninety random soil samples collected to a depth of 20 cm (primary active rooting depth in this location) and near the drip line of trees were pooled to form samples for six separate sub-areas within the trial and used for physiochemical analyses (Waters Agricultural Laboratories, Inc., Camilla, GA, USA). Results indicated organic matter content of 0.60% (S.D. = 0.12), pH of 5.03 (S.D. = 0.24), cation exchange capacity of 3.40 meq/100g (S.D. = 0.28), and a composition of 94.8% sand, 3.0% silt, and 2.2% clay.

**Figure 1:**
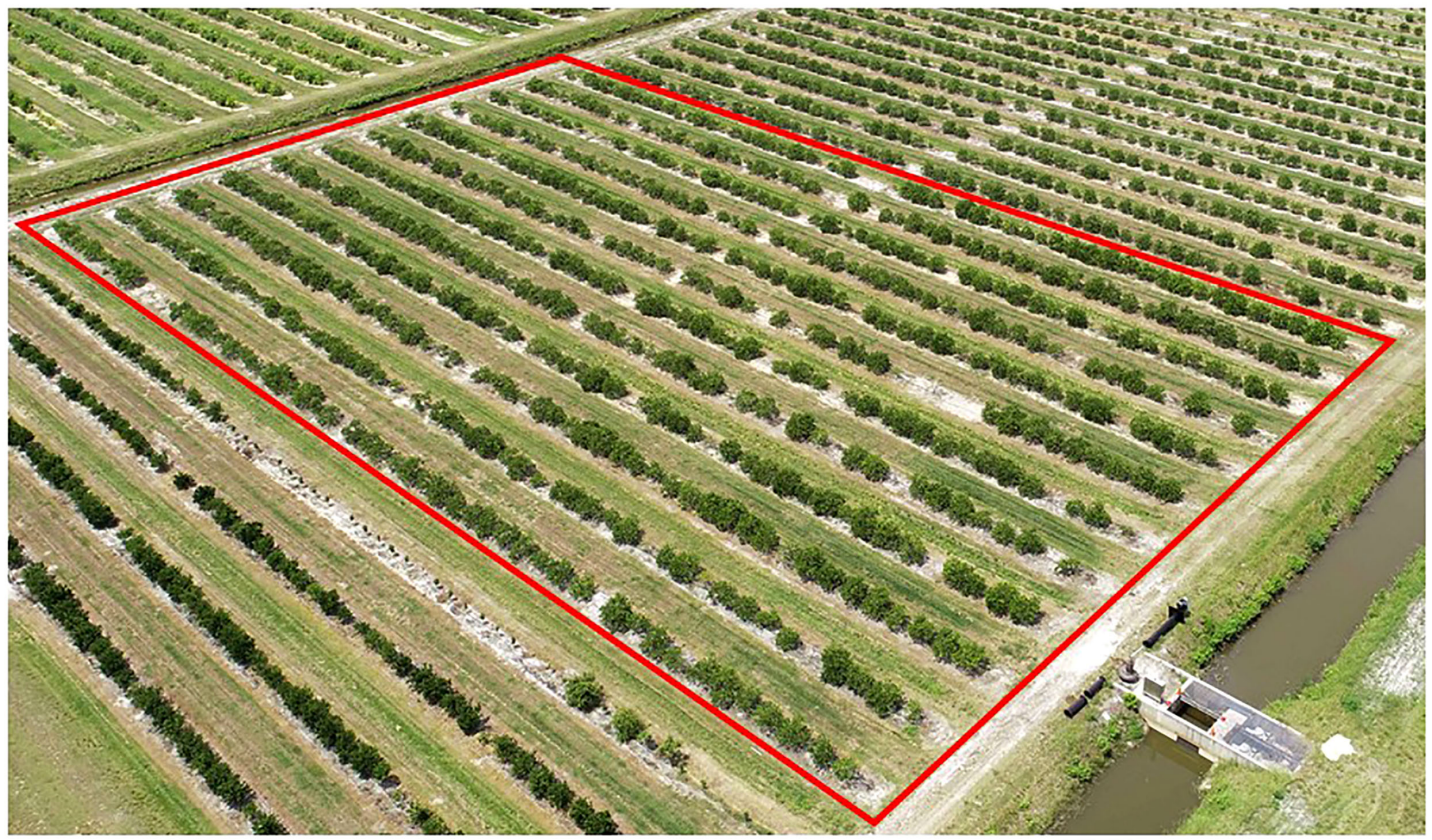
Aerial image of the Picos 2014 rootstock trial in 2021.

A total of 525 trees were planted as single tree replications into double row raised beds at 2.1 m × 7.6 m spacing, using a randomized design with approximately one replication of each rootstock in each of 12 rows (**Figure 1**). Most rows contained one replication of all of the 50 rootstocks. The number of replications/trees per rootstock in the trial ranged from 8-12. The experimental trial is at an average elevation of 7.9 m above sea level, with good drainage, although the area has a relatively high water table that restricts root development to less than 1 m depth. The experimental trial was irrigated by microjet when rainfall was insufficient to meet tree needs. Irrigation water in the trial is obtained from 18-37 m deep wells and considered good quality with total dissolved solids of 541 ppm, 58 ppm sodium, and pH of 7.5. Care of trees in the field during the trial employed common nutritional treatment with 4-5 applications per year of dry and slow-release fertilizers containing N, P, K and minors, following the University of Florida/IFAS recommended best management practices (https://edis.ifas.ufl.edu/publication/ss478). Weed, disease, and insect control was applied using typical drench and spray application of common pesticides. The experimental trial was surrounded on all sides by border trees that were not part of the experiment.

### Disease conditions

The area of the trial is affected by root rot caused by *Phytophthora nicotiana* and *Phytophthora palmivora*, as well as citrus canker (*Xanthomonas axonopodis*) and citrus tristeza virus (CTV). Tree health in the trial is considered most severely affected by huanglongbing disease (HLB), associated with *Candidatus* Liberibacter asiaticus (CLas), which has been classified as endemic in Florida. Previous experience with newly planted trees in this area indicates that most trees become infected by CLas within 2-3 years after planting to the field. A survey of a random sample of 12% of experimental trees in the trial during 2021 was completed to estimate the level of CLas and CTV infection in the trial, using methods for CLas and CTV detection by qPCR as previously described (Bowman and Albrecht, 2015; Bowman et al., 2016a).

### Tree survival and size

Tree survival was determined as the percent of originally planted trees for each rootstock that survived through the fourth harvest in 2021. Trees that died before fruit harvest in 2021 were not used in determination of other traits besides survival. For trees that survived, measurements were made of trunk diameter at 5 cm above and below the graft union in 2021 (both N-S and E-W directions) with a digital caliper, and used to calculate scion and rootstock trunk diameters, as well as scion/rootstock ratio. Relative growth differential between rootstock and scion (scion/rootstock ratio) provided an estimate of the potential for any associated graft union disruption from rootstock overgrowth, as has been previously suggested for some graft combinations on Swingle rootstock (Bowman and Joubert, 2020). Manual measurements were also made of canopy diameter (both N-S and E-W directions) and canopy height with tape measure and height pole. These were used to calculate canopy diameter and canopy volume using the formula volume = (diameter^2^ × height)/4, as previously described (Wutscher and Hill, 1995).

### Fruit crop

Fruit yield was determined in March 2018, 2019, 2020, and 2021 by making complete counts of fruit on each tree at harvest time and determining average weight per fruit from a fruit sample from each tree. Fruit yield rank was determined by comparison of cumulative fruit yield per tree over the four seasons of evaluation. Fruit yield efficiency was calculated as the average annual yield per tree during the 2020 and 2021 seasons divided by the canopy volume just before harvest in 2021.

### Fruit quality

Fruit quality for each replication each year was determined from a random sample of 12 fruit collected at harvest (March each year). Each fruit sample was weighed and juiced in a small sample auto-feed fruit juicer (FMC Multifruit Fresh N’squeeze juicer, model POS1; JBT FoodTech Citrus Systems, Lakeland, FL, USA). The weight of juice was determined and used to calculate percent juice. Juice samples were used for measurement of total soluble solids, total acids, and juice color. Total soluble solids was measured using a digital refractometer (RX-5000a, Atago Inc., Bellevue, WA, USA). Total acids was measured by automated titration (InMotion Max Autosampler SD660 and T50 Titrator pump; Mettler-Toledo, LLc, Columbus, OH, USA). Juice color was measured using a benchtop spectrophotometer (Color i5; x-Rite Pantone, Grand Rapids, MI, USA). Many trees did not have enough fruit for a quality analysis in 2018, so fruit quality results were determined as the average values for each trait in the three seasons 2019, 2020, and 2021.

### Tree health and HLB symptoms

Canopy health was determined on eight dates between 2018 and 2021 (1/2018, 2/2019, 5/2019, 2/2020, 12/2020, 4/2021, 8/2021, 10/2021) by visual assessment of all trees. Trees were rated on a scale of 1 to 5, with 1 representing the most yellow tree color and thinnest canopy, and 5 representing the best healthy green color and thickest canopy. Canopy health was a combined assessment of canopy color and canopy thickness in one value. Canopy health values were averaged for the time periods 2018-19 and 2020-21.

Canopy color and canopy thickness were separately determined by visual assessment of all trees in October 2021. Canopy color and canopy thickness were rated on a scale of 1 to 5, with 1 representing the worst and 5 representing the best. Four ratings of each trait per tree were performed by dividing the tree into a north and south half on each side of the row, and the average was calculated for each tree.

Foliar disease symptoms (HLB disease index) were determined by visual assessment of all trees in October 2021, using a scale of 1 to 5, with 1 representing the best (no foliar disease symptoms) and 5 representing the worst (75% to 100% of the canopy showing foliar disease symptoms); ratings of 2, 3, and 4 represented 1% to 25%, 25% to 50%, and 50% to 75%, respectively, of the affected canopy. HLB symptoms included blotchy mottling of leaves, chlorosis, and other abnormalities associated with HLB (Bove, 2006). Four ratings per tree were performed by dividing the tree into a north and south half on each side of the row, and the average was calculated for each tree.

Pre-mature fruit drop was determined for all trees in 2021 by doing a full count of fruit on each tree 4 weeks before harvest, and comparing that to the count of fruit on each tree at harvest.

Good association between different measures of tree health were observed, so an average tree health rank was calculated by averaging the rank of each rootstock in each of the six tree health categories.

### Optimum tree size calculations

Trees in the trial were planted at a density of 640 trees per hectare. Optimum spacing of trees in the row and between rows was determined by using tree diameter (at 6 years) for each rootstock as the optimum tree spacing within a row, and using tree diameter plus 2.44 m (estimated farm equipment width) as the optimum spacing between rows. The estimated optimum number of trees per hectare was calculated by the formula


Optimum trees per hectare=10,000/ [tree dia x(tree dia+2.44 m)]

The optimum number of trees per hectare for each rootstock was used to calculate optimum fruit yield (kg) and total soluble solids yield (kg) per hectare per season, based on the average fruit and total soluble solids yields on each rootstock for the 2020 and 2021 seasons.

### Estimation of variance components and trait heritability

Eight different parental combinations were represented by two or more hybrids in the trial and these families were used to evaluate the difference in parental influence on each trait. Pedigree-based variance estimation is a typical approach for partitioning additive genetic variance in full-sib/half-sib families, especially for unbalanced designs (Bernardo, 2020). In this trial, the intercrossing scheme was unstructured, and number of siblings evaluated per family varied. To account for the unbalanced design, the contribution of additive genetic variance to each trait was estimated first using restricted maximum-likelihood (REML) linear mixed models (Endelman, 2011). This was followed by a Bayesian approach using MCMCglmm (Hadfield, 2010).

### REML linear mixed model estimates of additive genetic variance

The phenotypic records were collated for 46 hybrids derived from intercrossing 16 parents resulting in 12 unique genetic combinations. Intercrosses resulting in fewer than two hybrids were excluded from further analysis as well as industry standard cultivars (Cleopatra, Swingle, sour orange, and Ridge). Analysis was restricted to 42 hybrids in 8 full-sib families that consisted of at least 2 members, with an average of 5.25 siblings per family and an average of 10.47 replications per hybrid (range = 8-12). Phenotypic values for each of the 31 measured traits were Box-Cox transformed with the bestNormalize package in R (Peterson, 2021). The following model was applied to estimate the contribution of additive genetic variation to each phenotype with the method mixed.solve() from the rrBLUP package (Endelman, 2011):


y=Xβ+Za+ϵ

where *y* is a vector of transformed phenotypes, β is a vector of fixed effects, a is vector of additive genetic effects, and ϵ is a vector of the random residual effects. *X* represents the fixed effects design matrix and Z the random effects design matrix. The pedigree-based additive relationship matrix was generated using getA() in the package pedigreemm (https://cran.r-project.org/web/packages/pedigreemm/index.html). Narrow-sense heritability *h* ^2^ was calculated as the $\frac{\sigma_{\text{a}}^{2}}{\sigma_{\text{a}}^{2}+\sigma_{\text{ϵ}}^{2}}$ , where $\sigma_{\text{a}}^{2}$ is the pedigree-based estimate of additive genetic variance and $\sigma_{\text{ϵ}}^{2}$ is the residual variance. The predicted estimated breeding values (EBV), or Best Linear Unbiased Predictors (BLUP), were also extracted from each trait model.

### MCMC generalized linear mixed model estimates of additive genetic variance

Pedigree-based analysis of trait variation using MCMCglmm was also restricted to the 42 hybrids in 8 full-sib families that consisted of at least 2 members. In this case, the pedigree and additive relationship matrix were generated using the package ‘nadiv’ (Wolak, 2012). Estimates of $\sigma_{\text{a}}^{2}$ and $\sigma_{\text{ϵ}}^{2}$ were obtained from MCMCglmm and narrow-sense heritability (*h* ^2^) was also calculated as $\frac{\sigma_{\text{a}}^{2}}{\sigma_{\text{a}}^{2}+\sigma_{\text{ϵ}}^{2}}$ . MCMCglmm also calculates confidence intervals (Highest Posterior Density Intervals, or HPD Intervals). Prior densities were set as V=1, nu=0.0002 following (Martínez-García et al., 2017). Posterior density estimates of unknown parameters were generated through a Monte Carlo Markov chain (MCMC) with the following parameters guiding the sampling process – iterations (nitt) of 100,000, burn-in of 10,000, and thinning interval of 10. Model convergence was determined if autocorrelation between iterations was less than 0.1 and effective size was greater than 1000. Three of the 31 traits did not reach convergence including fruit yield (2018), fruit color (2019-2021), and total soluble solids/acid ratio (2019-2021) based on autocorrelation, but effective sizes were greater than 1000.

### Statistical analysis

A one-way analysis of variance was conducted for all traits, and the comparison of means was performed by Tukey’s honestly significant difference test. Differences were defined as statistically significant when P< 0.05. Data were analyzed using Statistica version 14.0.0.15 (TIBCO Software, Palo Alto, CA, USA).

## Results

### Disease conditions

A survey of experimental trees in the trial during 2021 indicated 100% of trees were infected with CLas and 90% of trees were infected with CTV. In trees determined positive for each pathogen by real time PCR (Ct<38), values for Ct_CLas_ = mean 23.05, std. dev. 0.95, and values for Ct_CTV_ = mean 26.78, std. dev. 1.21.

### Tree survival and size

Among the 525 total experimental trees that were planted in 2014, 494 trees survived through the fourth harvest in 2021. Average percent survival for the 50 rootstocks was 94% (**Table 2**), with 35 rootstocks having no trees that died, including standard sour orange, Swingle, and Cleopatra. The rootstock that had the highest percent of tree death was Ridge sweet orange, with half of all trees on that rootstock dead by harvest in spring 2021.

**Table 2:** Tree survival, tree size, and scion/rootstock diameter ratio.

Rootstock	Percent tree survival (reps)	Scion trunk diameter^ab^ (mm)	Rootstock trunk diameter (mm)	Scion/Rootstock ratio	Canopy height (m)	Canopy diameter (m)	Canopy volume (m^3^)	Size rank and group^c^ (S, M, L)
Sour orange	100 (12)	95.1 a	106.1 a-c	0.897 ab	2.20 a	2.67 a-c	3.96 ab	1 L
US-1709	100 (10)	94.6 ab	107.3 a-c	0.882 a-d	2.15 ab	2.68 a-c	3.84 ab	2 L
US-1688	92 (12)	93.2 ab	106.1 a-c	0.878 a-e	2.11 a-c	2.81 a	4.26 a	3 L
US-1687	90 (10)	90.5 a-d	102.0 a-g	0.887 a-d	2.05 a-c,e	2.77 ab	3.97 ab	4 L
Cleopatra	100 (11)	90.0 a-c	104.7 a-d	0.864 a-f,h	1.99 a-f	2.47 a-f,h	3.04 b-d,f	5 L
US-1691	90 (10)	89.4 a-d	102.2 a-f	0.875 a-f	2.05 a-c,e	2.50 a-f	3.25 a-d	6 L
US-2152	100 (10)	86.3 a-e	109.5 ab	0.792 e-o	2.13 a-c	2.58 a-d	3.56 a-c	7 L
US-1653	90 (10)	85.0 a-e,g	99.3 b-h	0.857 a-h,j	1.92 a-g	2.58 a-d	3.33 a-d	8 L
US-1680	89 (9)	84.3 a-g	88.6 d-m	0.952 a	2.00 a-f	2.47 a-h	3.07 a-f	9 L
US-1649	91 (11)	84.1 a-e,g	101.7 b-f	0.827 b-l	1.98 a-f	2.42 a-i	2.92 b-g	10 L
US-1672	100 (10)	83.7 a-e,g	94.0 b-j	0.892 a-c	2.03 a-e	2.70 a-c	3.73 a-c	11 ML
US-2111	100 (11)	83.5 a-e,g	94.4 b-i	0.884 a-d	1.88 a-h	2.45 a-f,h	2.86 b-g,j	12 ML
US-2336	90 (10)	82.4 a-h	94.4 b-j	0.872 a-f	2.04 a-c,e	2.38 a-j	2.89 b-h,j	13 ML
US-1694	100 (10)	81.8 b-h	92.1 c-j,l	0.887 a-d	1.98 a-f	2.52 a-e	3.27 a-d	14 ML
US-2109	100 (9)	80.6 b-i	99.8 b-h	0.808 c-m	1.98 a-f	2.47 a-f,h	3.05 b-f	15 ML
US-2132	100 (11)	78.9 c-j	98.3 b-h	0.802 d-m	1.88 a-h	2.24 d-n	2.37 d-m	16 ML
US-2338	100 (8)	78.6 c-k	101.9 a-h	0.773 g-p	1.86 a-i	2.46 a-i	2.81 b-k	17 ML
Swingle	100 (11)	77.8 c-k	118.2 a	0.658 q	2.03 a-c,e	2.46 a-f,h	3.15 a-d	18 ML
US-1676	70 (10)	77.6 c-l	90.2 c-m	0.861 a-h,j	1.92 a-h	2.43 a-j	2.87 b-j	19 ML
US-2343	75 (12)	77.6 c-k	94.3 b-j	0.830 b-l	1.80 b-i	2.17 d-p	2.24 d-m	20 ML
US-1673	100 (10)	76.6 d-l	88.7 d-m	0.864 a-h	1.86 a-h	2.33 b-k	2.54 c-l	21 M
US-2143	100 (9)	74.0 e-m	89.4 d-l	0.827 b-m	1.75 c-j	2.17 d-p	2.10 d-n	22 M
US-1678	100 (9)	73.9 e-m	78.6 i-n	0.942 a	1.69 d-j	2.20 d-p	2.08 d-n	23 M
US-2137	100 (12)	73.6 e-m	86.7 f-m	0.853 b-h,j	1.70 d-j	2.29 c-l	2.23 d-m	24 M
US-1701	100 (11)	73.5 e-m	90.4 d-j,l	0.816 b-m	1.82 b-i	2.22 d-o	2.26 d-m	25 M
US-2257	100 (11)	73.1 e-m	86.2 f-m	0.847 b-h,j	1.78 b-i	2.32 c-k	2.38 d-m	26 M
US-2293	100 (9)	72.4 e-n	94.5 b-j	0.766 i-p	1.97 a-f	2.26 c-m	2.54 c-l	27 M
US-2173	100 (10)	72.2 f-n	103.5 a-e	0.711 n-q	1.89 a-h	2.33 b-k	2.59 c-l	28 M
US-2158	100 (10)	72.0 f-n	101.3 b-g	0.711 n-q	1.82 b-i	2.16 d-p	2.14 d-m	29 M
US-2104	100 (12)	71.7 f-n	94.2 b-i	0.762 i,k-p	1.67 d-j	2.09 f-p	1.91 e,g-n	30 M
US-1790	100 (10)	69.8 f,h-o	85.2 g-m	0.826 b-l	1.80 b-i	2.19 d-p	2.35 d-m	31 MS
US-2106	100 (10)	68.6 h-o	91.0 c-j,l	0.753 k-p	1.76 c-i	2.23 d-o	2.26 d-m	32 MS
US-2135	91 (11)	68.4 h-o	86.8 f-m	0.789 f-o	1.83 b-i	2.05 g-q	1.95 e-n	33 MS
US-2250	100 (11)	68.1 i-o	86.1 f-m	0.790 f-o	1.73 d-j	2.12 e-p	1.94 e-n	34 MS
US-2234	100 (11)	66.7 j-o	81.1 i-n	0.823 b-l	1.66 d,f-j	2.02 i-q	1.71 hi,k-n	35 MS
US-2272	100 (12)	66.0 k-p	89.2 e-j,l	0.739 m-q	1.75 d-i	2.11 e-p	1.97 e-n	36 MS
US-2107	100 (10)	65.6 j-p	78.1 j-n	0.840 b-k	1.67 d-j	2.02 g,i-q	1.72 h-n	37 MS
US-2214	100 (11)	65.3 k-p	77.7 k-n	0.840 b-k	1.68 d-j	2.04 g-i-q	1.78 g-n	38 MS
US-1681	100 (9)	64.6 k-p	84.2 h-m	0.767 i-p	1.71 d-j	2.06 f-q	1.84 e-n	39 MS
US-2280	100 (12)	63.9 l-p	79.3 i-n	0.804 d-m	1.67 f-j	1.99 j-q	1.67 i,k-n	40 MS
US-2156	100 (10)	62.2 m-p	79.2 i-n	0.786 f-o	1.65 f-j	1.93 k-q	1.54 k-o	41 S
US-2102	100 (11)	61.2 m-p	86.4 f-m	0.709 o-q	1.74 d-i	1.97 j-q	1.71 hi,k-n	42 S
Ridge	50 (12)	60.6 m-p	70.7 mn	0.855 a-j	1.48 h-k	1.82 m-q	1.35 l-o	43 S
US-2136	89 (9)	60.0 m-p	79.6 i-n	0.752 k-p	1.52 g-k	1.76 pq	1.22 m-o	44 S
US-2123	55 (11)	59.4 m-p	77.7 i-n	0.768 g,i-p	1.54 g-k	1.85 l-q	1.34 l-o	45 S
US-2240	91 (11)	58.2 op	72.9 k,mn	0.799 d-n	1.56 g-j	1.82 o-q	1.31 m-o	46 S
US-2229	64 (11)	57.7 n-p	77.8 i-n	0.741 l-q	1.70 d-j	1.84 l-q	1.45 i,k-o	47 S
US-2153	100 (12)	53.6 p	66.1 n	0.817 b-m	1.38 jk	1.64 qr	0.95 no	48 S
US-1679	100 (10)	53.1 p	76.9 k-n	0.690 pq	1.47 i-k	1.82 n-q	1.29 m-o	49 S
US-1103	100 (11)	38.9 q	47.5 o	0.832 b-l	1.14 k	1.23 r	0.46 o	50 S
*Average*	*94 (10.5)*	*73.2*	*89.8*	*0.816*	*1.81*	*2.22*	*2.38*	
*P-value*		*<0.00001*	*<0.00001*	*<0.00001*	*<0.00001*	*<0.00001*	*<0.00001*	

a Mean groups for significant ANOVA within columns were by Tukey test at P<0.05.

b Table is sorted by this column.

c Size rank groups: L, large; ML, medium-large; M, medium; MS, medium-small; S, small.

Tree size was strongly affected by rootstock, with significant rootstock effects on scion and rootstock trunk diameter, scion canopy height, scion canopy diameter, and canopy volume (**Table 2**). Sorting rootstocks by scion trunk diameter, the rootstock which induced the largest tree was standard sour orange with a scion trunk diameter of 95.1 mm, and the rootstock which induced the smallest tree was US-1103 (Mw × Chinotto) with a diameter of 38.9 mm. Rootstocks were ranked by influence on scion trunk diameter, and grouped into categories of large, medium-large, medium, medium-small, and small. Generally, categorization of rootstock influence on tree size was similar when compared by tree height, tree diameter, and canopy volume, although there were some discrepancies, such as was the case with trees on Cleopatra and Swingle, which had a smaller and larger canopy volume, respectively, than might have been anticipated based on scion trunk diameter. The rootstock creating the largest canopy volume in 2021 was US-1688 (Cm × Cleopatra) at 4.26 m^3^, while trees on US-1103 had the smallest canopy volume at 0.46 m^3^.

Rootstock trunk diameter also was associated with other measures of tree size, although it was used in this study primarily to assess relative growth differential between rootstock and scion and provide an estimate of the potential for any associated graft union disruption from rootstock overgrowth. In our study, Swingle had the largest growth differential between rootstock and scion (scion/rootstock ratio of 0.658), while standard sour orange, Cleopatra, and many of the hybrid rootstocks exhibited ratios approaching 1, indicating very little rootstock overgrowth relative to the scion. The hybrid rootstock US-1680 (Cm × Tachibana) had the smallest rootstock and scion growth differential (scion/rootstock ratio of 0.952).

### Crop size and yield efficiency

Fruit crop production per tree showed significant rootstock effects for each of the four seasons (2018-21), with the largest difference among rootstocks in 2021, the last season evaluated (**Table 3**). Rootstocks were ranked for influence on four-year cumulative fruit yield, resulting in rankings of 15, 21, 45, and 49 for standard sour orange, Swingle, Cleopatra, and Ridge, respectively. The three rootstocks with the highest cumulative yields were US-1688, US-2338, and US-1672, with values of 56.76, 49.82, and 46.67 kg per tree, respectively. The rootstocks US-1688 and US-1672 are both hybrids of Cm × Cleopatra, while US-2338 is a hybrid of Cm × (Changsha × Pt).

**Table 3:** Fruit yield per tree and average annual yield efficiency.

Rootstock	2018 fruit yield^a^ (kg)	2019 fruit yield (kg)	2020 fruit yield (kg)	2021 fruit yield (kg)	Cumulative fruit yield 2018-21^b^ (kg)	Fruit yield rank	2020-21 annual yield efficiency (kg/m^3^)
US-1688	5.52 a-c	16.72 a	15.61 b	18.90 a	56.76 a	1	4.16 a-e
US-2338	5.89 a-c	11.69 a-d	15.76 ab	16.47 a-d	49.82 ab	2	5.91 ab
US-1672	8.31 ab	9.93 a-e	14.72 ab,d	13.71 a-g,i	46.67 a-c	3	3.76 a-f
US-1709	4.53 a-c	11.14 a-d	14.28 a-d	16.57 a-c	46.52 a-c	4	3.99 a-e
US-1649	5.77 a-c	10.30 a-e	13.77 a-e	16.53 a-c	46.38 a-c	5	5.38 a-d
US-1676	7.76 a-c	7.33 a-f	13.56 a-f	17.54 ab	46.19 a-d	6	5.50 a-d
US-2152	6.49 a-c	14.01 ab	11.20 a-f	11.60 a-k	43.31 a-e	7	3.30 b-f
US-2111	4.83 a-c	11.90 a-c	14.20 ab,d	11.11 b-k	42.05 a-e	8	4.58 a-e
US-1680	5.30 a-c	11.68 a-d	11.83 a-f	13.06 a-i	41.87 a-f	9	4.17 a-e
US-1694	6.38 a-c	7.90 b-f	12.76 a-f	14.75 a-f	41.79 a-f	10	4.27 a-e
US-1687	2.71 a-c	9.62 a-e	11.65 a-f	15.78 a-e	39.76 a-h	11	3.39 a-f
US-2336	6.67 a-c	12.24 a-c	10.53 a-f	10.09 b-k	39.54 a-h	12	3.68 a-f
US-1673	8.49 a	6.87 b-f	12.66 a-f	11.47 a-k	39.49 a-g	13	4.80 a-e
US-1678	6.32 a-c	8.10 a-f	12.24 a-f	12.16 a-j	38.82 a-i	14	5.60 a-d
US-2109	7.34 ab	11.45 a-d	8.51 a-g	11.25 a-k	38.55 a-i	15	3.21 b-f
US-2137	5.90 a-c	7.79 b-f	10.84 a-f	13.50 a-g	38.04 a-h	16	5.51 a-c
Sour orange	3.67 a-c	5.74 b-f	14.40 ab,d	13.52 a-g,i	37.32 a-i	17	3.59 a-f
US-2293	6.55 a-c	9.67 a-e	11.58 a-f	9.51 b-k	37.30 a-j	18	4.03 a-f
US-1691	4.50 a-c	7.21 b-f	8.67 a-g	15.94 a-e	36.32 a-j	19	3.73 a-f
US-1653	5.32 a-c	8.56 a-f	9.04 a-g	12.98 a-g,i	35.91 a-j	20	3.27 b-f
Swingle	3.78 a-c	9.35 a-e	10.38 a-f	10.96 b-k	34.47 b-k	21	3.40 b-f
US-1681	5.88 a-c	7.50 b-f	9.15 a-g	10.16 b-k	32.69 b-k	22	5.50 a-d
US-2257	5.50 a-c	7.65 b-f	9.81 a-g	9.60 b-k	32.57 b-k	23	3.98 a-e
US-2250	6.55 a-c	6.15 b-f	9.56 a-g	9.50 b-k	31.76 b-k	24	4.79 a-e
US-2343	4.45 a-c	6.14 b-f	10.74 a-f	10.40 b-k	31.73 b-k	25	4.85 a-e
US-2143	2.76 a-c	7.65 b-f	12.13 a-f	8.57 b-l	31.12 b-k	26	5.01 a-e
US-2132	6.09 a-c	6.87 b-f	7.98 a-g	10.10 b-k	31.05 b-k	27	3.76 a-f
US-2280	6.69 a-c	7.18 b-f	6.78 a-g	8.93 b-k	29.58 b-k	28	4.74 a-e
US-1790	2.94 a-c	5.37 b-f	10.45 a-f	9.78 b-k	28.55 b-k	29	3.87 a-f
US-2104	6.54 a-c	6.87 b-f	8.64 a-g	6.05 h-l	28.10 c-k	30	3.96 a-e
US-2135	2.54 a-c	6.26 b-f	8.38 a-g	10.26 b-k	27.43 b-k	31	4.73 a-e
US-2234	6.78 a-c	5.84 b-f	7.24 a-g	7.56 f-l	27.42 c-k	32	4.50 a-e
US-2106	4.40 a-c	6.54 b-f	9.30 a-g	5.81 g-l	26.05 c-k	33	3.46 a-f
US-1701	3.83 a-c	4.61 c-f	7.30 a-g	8.68 c-k	24.42 d-k	34	3.56 a-f
US-2272	3.09 a-c	5.00 c-f	7.78 a-g	8.47 d-k	24.34 d-k	35	4.09 a-e
US-1679	5.68 a-c	4.12 c-f	5.78 a,c-g	8.17 d-l	23.76 d-k	36	5.68 ab
US-2123	3.20 a-c	2.83 c-f	9.98 a-g	6.98 e-l	22.99 d-l	37	6.50 a
US-2158	3.59 a-c	5.60 b-f	5.74 a,c-g	6.42 g-l	21.35 f-l	38	2.71 d-f
US-2136	5.66 a-c	5.90 b-f	4.58 c-g	5.14 h-l	21.28 e-l	39	4.06 a-f
US-2173	3.50 a-c	4.02 c-f	5.66 a,c-g	6.18 g-l	19.37 g-l	40	2.35 ef
US-2214	2.42 a-c	4.99 c-f	5.94 a,c-g	6.01 g-l	19.35 g-l	41	3.34 b-f
US-2240	3.96 a-c	3.39 c-f	5.65 a,c-g	5.82 g-l	18.81 g-l	42	4.45 a-e
US-2156	5.03 a-c	2.32 d-f	5.36 a,c-g	5.65 h-l	18.36 g-l	43	3.54 a-f
US-2102	5.30 a-c	2.12 ef	4.46 e-g	6.35 g-l	18.23 i-l	44	3.03 b-f
Cleopatra	2.00 bc	2.51 d-f	6.01 a,c-g	7.34 f-l	17.86 i-l	45	2.18 ef
US-2153	5.15 a-c	2.53 ef	4.88 c,e-g	4.71 j-l	17.27 j-l	46	4.55 a-e
US-2107	2.28 a-c	3.89 c-f	4.56 c,e-g	5.69 h-l	16.43 j-l	47	2.84 c-f
US-2229	2.47 a-c	2.89 c-f	7.05 a-g	3.66 h,j-l	16.07 h-l	48	3.51 a-f
Ridge	3.21 a-c	3.25 c-f	2.75 fg	2.63 kl	11.83 kl	49	2.05 ef
US-1103	0.77 c	0.44 f	0.45 g	0.64 l	2.30 l	50	1.17 f
*Average*	*4.89*	*6.99*	*9.25*	*9.85*	*30.89*		*4.04*
*P-value*	*<0.00001*	*<0.00001*	*<0.00001*	*<0.00001*	*<0.00001*		*<0.00001*

a Mean groups for significant ANOVA within columns were by Tukey test at P<0.05.

b Table is sorted by this column.

Average annual yield efficiency (during the 2020 and 2021 seasons) also demonstrated significant rootstock effects (**Table 3**). The rootstock US-2123 produced the highest yield efficiency at 6.50 kg/m^3^ because of small tree size (ranked 45^th^ in tree size), even though it was ranked 37^th^ in cumulative yield. The rootstock US-2338 was second highest in yield efficiency at 5.91 kg/m^3^, as a result of very high fruit production on a medium-large size tree. The rootstocks US-1103 and Ridge were the two lowest in yield efficiency, despite small tree size, as a result of very low cumulative yield.

### Fruit quality

The fruit quality traits total soluble solids (TSS; %), total acid (%), percent juice, juice color (CN), individual fruit weight (g), and total soluble solids (kg) per metric ton (MT) of fruit all exhibited significant rootstock effects (**Table 4**). Only the trait TSS:acid ratio was not observed to show significant rootstock effects.

**Table 4:** Fruit quality for the 2019-21 harvest seasons.

Rootstock	Total soluble solids^ab^ (%)	Total acid (%)	TSS : Acid ratio	Juice (%)	Juice color (CN)	Fruit weight (g)	Soluble solids per MT (kg)
US-2343	8.53 a	0.776 a-e	11.26	56.24 a	36.93 ab	176 a-e	48.01 a
US-1103	8.50 a-g	0.786 a-f	11.03	54.05 a-c	37.16 ab	145 de	45.82 a-h
US-2104	8.43 ab	0.770 a-e	11.06	55.40 a-c	36.83 ab	177 a-e	46.74 ab
US-2152	8.36 a-c	0.797 a-d	10.58	53.69 a-c	36.81 ab	154 de	44.88 a-f
US-2123	8.35 a-e	0.821 a-c	10.47	54.15 a-c	36.89 ab	181 a-e	45.19 a-f
US-2173	8.35 a-e	0.743 a-f	11.62	54.57 a-c	36.82 ab	149 e	45.56 a-e
US-2102	8.29 a-f	0.729 a-f	11.51	55.29 a-c	36.95 ab	165 b-e	45.89 a-d
US-2257	8.29 a-f	0.722 a-f	11.64	54.12 a-c	36.92 ab	175 a-e	44.88 a-f
US-2153	8.29 a-d	0.833 ab	10.43	54.64 a-c	36.89 ab	171 a-e	45.39 a-e
US-2132	8.29 a-f	0.857 a	9.92	55.77 ab	36.92 ab	159 b-e	46.22 a-c
US-2136	8.25 a-h	0.809 a-d	10.36	53.86 a-c	36.88 ab	166 a-e	44.40 a-h
US-2214	8.04 a-i	0.688 c-f	11.84	53.82 a-c	36.96 ab	177 a-e	43.46 a-h
US-2143	8.03 a-i	0.725 a-f	11.29	55.12 a-c	37.02 ab	178 a-e	44.27 a-h
US-2250	7.99 a-i	0.711 b-f	11.52	54.68 a-c	37.03 a	172 a-e	43.72 a-h
US-2111	7.99 a-i	0.741 a-f	10.92	55.83 ab	36.89 ab	183 a-d	44.62 a-g
US-2280	7.97 a-i	0.753 a-f	10.85	54.71 a-c	36.91 ab	172 a-e	43.72 a-h
US-2293	7.96 a-i	0.748 a-f	10.88	52.80 a-c	36.72 ab	178 a-e	42.06 a-h
US-2137	7.96 a-i	0.714 b-f	11.23	54.87 a-c	37.03 a	183 a-d	43.69 a-h
US-1790	7.96 a-i	0.758 a-f	10.67	54.41 a-c	36.87 ab	156 c-e	43.30 a-h
US-2229	7.93 a-i	0.643 d-f	12.45	53.62 a-c	36.91 ab	182 a-e	42.58 a-h
US-2109	7.92 a-i	0.763 a-f	10.57	54.49 a-c	36.85 ab	170 a-e	43.17 a-h
US-2272	7.88 a-i	0.696 c-f	11.61	55.14 a-c	37.08 a	186 a-c	43.50 a-h
US-1676	7.84 a-i	0.704 b-f	11.27	53.53 a-c	36.78 ab	183 a-d	42.00 a-h
US-2107	7.83 a-i	0.751 a-f	10.70	53.05 a-c	36.89 ab	159 b-e	41.58 b-h
US-2156	7.81 a-i	0.752 a-f	10.60	53.97 a-c	36.81 ab	156 c-e	42.19 a-h
US-2135	7.78 a-i	0.782 a-e	10.14	54.71 a-c	36.75 ab	175 a-e	42.56 a-h
US-1673	7.78 a-i	0.712 b-f	11.04	53.49 a-c	36.96 ab	188 ab	41.62 b-h
US-2158	7.75 a-i	0.714 b-f	10.98	53.39 a-c	36.74 ab	156 de	41.36 b-h
Swingle	7.73 a-i	0.731 a-f	10.85	54.48 a-c	36.96 ab	166 b-e	42.17 a-h
US-2106	7.68 a-i	0.722 a-f	10.82	53.19 a-c	36.81 ab	165 b-e	40.83 c-h
US-1681	7.67 a-i	0.707 b-f	11.09	53.87 a-c	36.64 ab	183 a-d	41.30 b-h
US-1709	7.67 a-i	0.702 b-f	11.00	53.18 a-c	36.77 ab	175 a-e	40.80 c-h
US-2336	7.64 a-i	0.652 d-f	11.84	52.02 c	36.68 ab	183 a-d	39.74 d-h
Ridge	7.61 a-i	0.690 a-f	11.46	52.50 a-c	36.63 ab	171 a-e	39.87 b-h
US-1688	7.60 b-i	0.690 c-f	11.15	53.48 a-c	36.84 ab	176 a-e	40.72 c-h
US-1649	7.60 b-i	0.680 c-f	11.40	53.59 a-c	36.57 ab	199 a	40.74 c-h
US-1701	7.56 c-i	0.718 b-f	10.71	52.97 a-c	36.67 ab	173 a-e	40.09 d-h
US-2338	7.52 b-i	0.684 c-f	11.12	53.72 a-c	36.96 ab	191 ab	40.39 c-h
US-2240	7.51 c-i	0.736 a-f	10.44	53.10 a-c	36.80 ab	164 b-e	39.91 d-h
US-1679	7.47 c-i	0.658 d-f	11.57	52.98 a-c	36.77 ab	190 ab	39.55 e-h
US-1687	7.45 c-i	0.685 c-f	11.05	53.46 a-c	36.85 ab	168 a-e	39.84 d-h
US-1678	7.44 d-i	0.667 d-f	11.39	53.75 a-c	36.81 ab	183 a-d	40.00 d-h
US-1694	7.43 e-i	0.707 b-f	10.61	54.37 a-c	36.78 ab	182 a-d	40.44 c-h
US-1672	7.43 e-i	0.688 c-f	10.97	53.73 a-c	36.82 ab	178 a-e	39.92 d-h
US-1653	7.41 d-i	0.712 a-f	10.57	53.63 a-c	36.69 ab	183 a-d	39.73 d-h
US-2234	7.40 g-i	0.665 d-f	11.38	52.12 c	36.94 ab	180 a-d	38.71 h
US-1680	7.37 f-i	0.676 c-f	11.10	52.48 bc	36.73 ab	180 a-e	38.72 f-h
US-1691	7.34 g-i	0.703 b-f	10.60	53.76 a-c	36.86 ab	179 a-e	39.46 e-h
Cleopatra	7.33 hi	0.648 ef	11.46	52.99 a-c	36.48 b	165 b-e	38.83 gh
Sour orange	7.28 i	0.635 f	11.87	53.20 a-c	36.56 ab	183 a-d	38.73 h
*Average*	*7.83*	*0.723*	*11.06*	*53.92*	*36.84*	*174*	*42.26*
*P-value*	*<0.00001*	*<0.00001*	*0.08181*	*0.00002*	*0.00355*	*<0.00001*	*<0.00001*

a Mean groups for significant ANOVA within columns were by Tukey test at P<0.05.

b Table is sorted by this column.

A key fruit quality trait for juice orange crops is juice total soluble solids, because the fruit value is primarily calculated by how much total soluble solids (sugar) is recovered during juicing. Therefore, the rootstocks are sorted in **Table 4** by this trait. The rootstock US-2343 [Cm × (Changsha × Pt)] induced the highest TSS (8.53%), and highest percent juice (56.24%). Standard sour orange and Cleopatra induced the lowest TSS of the 50 rootstocks in the trial at 7% and 6% lower than the trial mean, respectively, as well as the lowest total juice acid levels at 12% and 10% lower than the trial mean, respectively. The rootstocks with the highest total acids concentration were US-2132 [Cm × (Sunki × Pt)] and US-2153 [Sunki × (Cm × Pt)], with acid values 19% and 15% higher than the trial mean, respectively. The rootstocks resulting in the lowest percent juice content were US-2234 (Shunkokan × Cm) and US-2336 [Cm × (Changsha × Pt)], which had values 3-4% lower than the trial mean.

Although significant, the range of rootstock influence on juice color was relatively small, with the rootstocks standard sour orange and Cleopatra exhibiting the lowest CN values at 0.8-1.0% lower than the trial mean. The rootstocks US-2137 [Cm × (Sunki × Pt)], US-2250 (Shunkokan × Cm), and US-2272 (Shunkokan × Cm) induced the highest juice color values, at 0.5-0.6% higher than the trial mean. Average fruit size is of less importance for juice oranges than for fresh market oranges, but this trait showed strong rootstock effects. The rootstocks US-1649 (Cm × Sunki) and US-2338 [Cm × (Changsha × Pt)] induced the largest fruit size at 10-14% larger than the trial mean, while the smallest fruit sizes were on the rootstocks US-1103 and US-2173 [Sunki × (Cm × Pt)] at 14-17% smaller than the trial mean.

In the value total soluble solids per MT, which is of key importance for juice oranges, Swingle induced a value (42.17 kg) close to the trial mean of 42.26 kg, while values were quite low for trees on standard sour orange, Cleopatra, and Ridge at 6-8% lower than the trial mean. The highest value of total soluble solids per MT was produced by fruit on trees with the rootstock US-2343 [Cm × (Changsha × Pt)] at 48.01 kg, or 14% higher than the trial mean.

### Canopy health and HLB symptoms

The six measures of canopy health and HLB symptoms all demonstrated significant rootstock effects (**Table 5**). In the rating of canopy health (combining canopy thickness and color) over the first and second half of the four-year period 2018-21, the rootstocks US-1688 (Cm × Cleopatra) and US-1709 (Cm × Shekwasha) were the best, with values of 4.03 and 4.07, respectively, in 2018-19, and with values of 4.58 and 4.44, respectively, in 2020-21. Trees of the rootstock US-1688 were also the best for the traits 2021 canopy color and 2021 canopy thickness, and the second best for the trait 2021 HLB symptoms.

**Table 5:** Canopy health and HLB symptoms.

Rootstock	2018-19 Canopy health^a^	2020-21 Canopy health	2021 Canopy color	2021 Canopy thickness	2021 HLB symptoms	2021 Pre-harvest fruit drop (%)	Mean rank^b^
US-1688	4.03 ab	4.58 a	3.93 a	4.30 a	2.25 de	28.2 e-g	1.7
US-1687	3.70 a-d	4.36 a-c	3.92 ab	4.00 ab	2.42 c-e	32.3 d-g	4.2
US-2338	3.58 a-e	4.05 a-g	3.78 a-d	3.69 a-e	2.13 e	30.4 d-g	4.5
US-1709	4.07 a	4.44 ab	3.73 a-e	3.98 ab	2.43 c-e	38.6 c-g	5.2
US-2152	3.73 a-d	4.00 a-g	3.63 a-g	3.68 a-e	2.40 c-e	37.1 c-g	7.3
US-1691	3.41 a-e	4.04 a-g	3.64 a-g	3.86 a-c	2.50 c-e	22.6 g	7.5
US-1672	3.83 a-c	4.04 a-f	3.63 a-g	3.75 a-d	2.43 c-e	39.7 b-g	8.2
US-1694	3.23 a-e	4.16 a-d	3.70 a-f	3.63 a-e	2.40 c-e	39.6 b-g	10.2
Swingle	3.30 a-e	3.95 a-h	3.77 ab,d	3.43 b-g	2.30 de	44.2 a-g	11.5
US-2109	3.63 a-d	3.80 b-i	3.47 a-h	3.56 a-f	2.67 a-e	43.3 a-g	13.0
US-1653	3.15 a-f	3.93 a-i	3.50 a-h	3.44 b-g	2.53 b-e	39.6 a-g	14.7
US-1676	3.43 a-e	3.60 c-k	3.32 a-j	3.11 c-h	2.68 a-e	22.9 fg	15.3
US-1790	3.07 b-f	3.60 d-k	3.65 a-g	3.43 b-g	2.50 c-e	38.8 b-g	15.7
US-1673	3.20 a-e	3.58 d-k	3.45 a-h	3.25 b-h	2.68 b-e	47.6 a-g	20.3
US-1701	3.21 a-e	3.62 d-k	3.41 a-i	3.23 c-h	2.75 a-e	48.0 a-g	21.0
Sour orange	3.11 b-e	4.05 a-e	3.40 a-i	3.38 b-g	2.85 a-e	49.6 a-g	21.7
US-2137	3.42 a-e	3.58 d-k	3.06 g-j	3.19 c-h	2.92 a-e	35.1 d-g	21.8
US-2272	3.22 a-e	3.53 d-k	3.29 b-j	3.19 c-h	2.60 b-e	53.1 a-g	22.7
US-2153	3.06 c-f	3.25 j-l	3.54 a-g	3.38 b-g	2.29 de	52.2 a-g	23.5
US-2135	3.30 a-e	3.48 d-k	3.10 c,e-j	3.05 d-h	2.70 a-e	37.4 c-g	23.7
US-2234	3.36 a-e	3.49 d-k	3.18 c-j	3.18 c-h	2.57 b-e	57.0 a-e	24.2
US-2257	3.33 a-e	3.38 f-k	3.16 c-j	3.16 c-h	2.66 b-e	51.0 a-g	24.2
US-2293	3.22 a-e	3.60 d-k	3.22 b-j	3.11 c-h	2.81 a-e	49.8 a-g	24.8
US-1649	3.50 a-e	3.52 d-k	3.10 c,e-j	2.95 e-h	2.95 a-e	39.3 b-g	25.3
US-2143	3.44 a-e	3.58 d-k	3.06 e-j	3.00 d-h	2.67 a-e	56.3 a-f	26.5
US-2250	3.09 b-f	3.42 e-k	3.20 c-j	3.07 d-h	2.84 a-e	42.4 a-g	27.7
US-2336	3.44 a-e	3.56 d-k	3.03 f-j	3.08 c-h	3.08 a-d	46.3 a-g	28.2
US-2106	3.37 a-e	3.36 f-k	3.23 c-j	3.10 c-h	2.78 a-e	60.8 a-d	28.5
Cleopatra	2.94 c-f	3.67 c-k	3.25 c-j	3.16 c-h	2.89 a-e	60.6 a-d	30.0
US-1680	3.33 a-e	3.50 d-k	2.97 g-j	3.13 c-h	3.22 a-c	51.0 a-g	30.8
US-2156	3.07 b-f	3.34 g-k	3.15 c-j	3.10 c-h	2.73 a-e	56.3 a-f	31.7
US-2229	3.38 a-e	3.23 h-l	3.07 c-j	2.89 e-h	2.64 a-e	74.4 a	32.0
US-2111	3.52 a-e	3.56 d-k	2.80 ij	2.86 f-h	3.25 a-c	51.4 a-g	32.5
US-2173	2.90 c-f	3.36 f-k	3.23 c-j	2.98 e-h	2.73 a-e	57.5 a-e	32.8
US-2132	3.30 a-e	3.24 j-l	2.91 h-j	2.82 f-h	3.23 a-c	30.0 e-g	33.0
Ridge	2.48 ef	3.17 h-l	3.25 a-j	2.96 d-h	2.42 b-e	69.1 a-c	33.2
US-2102	3.06 c-f	3.22 j-l	3.20 c-j	2.95 e-h	2.75 a-e	52.1 a-g	33.5
US-2280	3.11 b-e	3.15 j-l	3.06 g-j	2.85 f-h	2.81 a-e	45.3 a-g	34.3
US-2240	2.87 c-f	3.16 j-l	3.13 c,e-j	3.08 d-h	2.75 a-e	52.1 a-g	35.0
US-2104	3.08 c-f	3.03 kl	3.17 c-j	2.58 h	2.71 b-e	52.1 a-g	35.8
US-1678	3.00 c-f	3.29 h-l	2.97 g-j	2.81 f-h	3.08 a-d	42.3 a-g	36.5
US-1681	3.30 a-e	3.29 h-l	2.72 j	2.78 f-h	3.58 a	43.0 a-g	36.8
US-2158	2.87 c-f	3.26 i-l	3.08 e-j	3.00 e-h	2.93 a-e	54.3 a-g	37.7
US-2214	3.00 c-f	3.29 h-k	3.05 g-j	2.80 gh	2.70 b-e	59.9 a-d	37.7
US-2123	2.87 c-f	2.50 lm	3.08 a-j	2.83 c-i	2.83 a-e	50.9 a-g	38.3
US-2136	3.13 a-f	3.03 kl	2.97 g-j	2.56 hi	3.03 a-e	46.7 a-g	39.5
US-2343	3.00 c-f	3.00 kl	2.93 g-j	2.64 gh	3.00 a-e	44.4 a-g	40.3
US-1679	3.10 a-f	3.06 kl	2.73 j	2.55 h	3.40 ab	44.6 a-g	41.2
US-2107	2.83 d-f	3.16 j-l	3.00 g-j	2.83 f-h	3.03 a-e	55.0 a-f	42.5
US-1103	2.18 f	2.15 m	2.80 ij	1.80 i	2.84 a-e	73.2 ab	47.0
Mean	3.24	3.50	3.25	3.14	2.75	47.0	
*P-value*	*<0.00001*	*<0.00001*	*<0.00001*	*<0.00001*	*<0.00001*	*<0.00001*	

a Mean groups for significant ANOVA within columns were by Tukey test at P<0.05.

b Table is sorted by this column. Mean rank is the average rank of that rootstock across the six prior columns associated with tree health and HLB symptoms. In each column, a rank “1” was given to the rootstock with the value indicating the best tree health, continuing to rank “50” which indicates the rootstock with the worst tree health. Note that for canopy health, canopy color, and canopy thickness the larger numeric value represents a healthier tree. For HLB symptoms and pre-harvest fruit drop, a smaller numeric value represents a healthier tree.

Pre-harvest fruit drop in 2021 ranged from a high of 74.4% of the crop for trees on rootstock US-2229 (Shunkokan × Cm), to a low of 22.6% for trees on the rootstock US-1691 (Cm × Cleopatra). The standard rootstocks had relatively high values for this trait, with 2021 pre-harvest fruit drop for Swingle, standard sour orange, Cleopatra, and Ridge at 44.2, 49.6, 60.6, and 69.1%, respectively.

There was a general similarity of rank within each rating of health and HLB symptoms, so an average of the ranks for each of the six health traits was calculated for each rootstock, providing values of 1.7 to 47.0. Standard sour orange was intermediate in average tree health rank (21.7), while Swingle was better (11.5), and Cleopatra (30.0) and Ridge (33.2) were worse. The rootstocks with the best average tree health rank were US-1688 and US-1687 (both hybrids of Cm × Cleopatra), and US-2338 [Cm × (Changsha × Pt)], with values of 1.7, 4.2, and 4.5, respectively.

### Production per hectare

Relative production of trees per hectare per year on the different rootstocks differed substantially according to whether the calculation was based on fruit production or total soluble solids production, and whether tree density was fixed at the density used in the trial or was adjusted according to what appeared to be optimum based on tree size (**Table 6**). The trial was planted at 640 trees per hectare, and calculated optimum densities for the different rootstocks ranged from 686 to 2334 trees per hectare.

**Table 6:** Annual fruit production per hectare at trial density (640 trees per hectare) and at calculated optimum density for each rootstock.

Rootstock	Fruit per hectare at 640 trees^a^ (kg)	Rank of fruit per hectare at 640 trees	Solids per hectare at 640 trees (kg)	Rank of solids per hectare at 640 trees	Optimum density (trees per hectare)	Fruit per hectare at optimum density (kg)	Rank of fruit per hectare at optimum density	Solids per hectare at optimum density^b^ (kg)	Rank of solids per hectare at optimum density
US-2338	10316 ab	2	414 ab	2	836	13536 a	1	544 a	1
US-1649	9698 a-c	5	389 a-c	5	856	13053 a	2	525 a	2
US-1676	9951 a-d	3	400 a-d	3	853	13005 ab	3	523 ab	3
US-2123	5427 b-l	30	252 a-i	28	1297	10533 a-f	11	485 a-c	4
US-2137	7791 a-i	14	333 a-g	11	932	11247 a-d	6	479 ab	5
US-1688	11044 a	1	440 a	1	686	11625 a-c	4	465 a-c	6
US-2343	6765 a-k	19	307 a-i	15	1051	10054 a-f	16	458 a-c	7
US-2111	8099 a-h	10	351 a-e	8	846	10557 a-e	10	458 a-c	8
US-1709	9873 ab	4	399 ab	4	732	11234 a-e	7	455 a-c	9
US-1678	7808 a-i	13	313 a-h	14	1001	11393 a-e	5	454 a-c	10
US-2143	6623 a-k	21	288 a-i	18	1014	10237 a-e	14	444 a-c	11
US-1673	7723 a-i	15	315 a-h	12	920	10789 a-e	8	439 a-c	12
US-1694	8802 a-f	8	354 a-e	6	825	10757 a-e	9	432 a-d	13
US-2250	6099 b-k	27	266 a-i	24	1052	9650 a-f	21	421 a-d	14
US-1681	6180 b-k	26	248 a-i	30	1094	10457 a-e	12	420 a-d	15
US-2293	6748 a-k	20	287 a-i	19	952	9671 a-f	20	409 a-d	16
US-2135	5964 b-k	28	250 a-i	29	1112	9767 a-f	18	406 a-d	17
US-1680	7964 a-i	11	306 a-i	16	830	10344 a-e	13	398 a-d	18
Sour orange	8935 a-e	7	343 a-f	9	732	10193 a-e	15	390 a-d	19
US-1672	9096 a-e	6	352 a-f	7	728	10019 a-e	17	387 a-d	20
US-2132	5788 b-k	29	265 a-i	26	964	8448 a-f	30	386 a-d	21
US-2152	7297 a-j	16	314 a-h	13	787	8948 a-f	24	385 a-d	22
US-1790	6475 a-k	23	281 a-i	20	1204	8799 a-f	26	381 a-d	23
US-2257	6214 b-k	25	273 a-i	23	911	8627 a-f	29	379 a-d	24
US-1691	7875 a-i	12	305 a-i	17	824	9745 a-f	19	374 a-d	25
US-2280	5027 d-k	33	213 b-i	33	1153	8814 a-f	25	373 a-d	26
US-1687	8778 a-g	9	342 a-h	10	702	9347 a-f	22	362 a-d	27
US-2336	6599 a-k	22	259 a-i	27	875	9051 a-f	23	355 a-d	28
US-2104	4699 e-l	36	218 b-i	31	1091	7594 a-f	36	355 a-d	29
Swingle	6829 a-k	18	274 a-i	21	846	8688 a-f	28	349 a-d	30
US-2272	5200 c-k	31	215 b-i	32	1054	8381 a-f	31	346 a-d	31
US-1679	4467 e-l	37	173 d-j	39	1347	8747 a-f	27	341 a-d	32
US-2109	6322 a-k	24	266 a-i	25	837	7991 a-f	34	334 a-d	33
US-2234	4735 e-l	35	185 c-j	36	1140	8305 a-f	32	324 a-d	34
US-1653	7048 a-k	17	273 a-i	22	800	8114 a-f	33	314 a-e	35
US-1701	5114 c-k	32	197 b-j	34	985	7659 a-f	35	296 a-e	36
US-2153	3068 j-l	48	137 h-j	46	1554	6595 b-g	40	295 a-e	37
US-2214	3823 h-l	40	175 d-j	38	1116	6344 c-g	43	286 a-e	38
US-2240	3669 h-l	42	140 f-j	44	1315	7379 a-f	37	283 a-e	39
US-2106	4834 d-l	34	188 b-j	35	989	7169 a-f	38	277 a-e	40
US-2156	3524 h-l	43	148 e-j	43	1198	6499 b-g	41	274 a-e	41
US-2136	3111 i-l	47	133 e-j	48	1374	6437 b-g	42	274 a-e	42
US-2229	3429 g-l	45	138 e-j	45	1279	6639 a-g	39	269 a-e	43
US-2102	3459 i-l	44	153 e-j	42	1166	5988 c-g	44	264 a-e	44
US-2173	3789 h-l	41	176 d-j	37	911	5335 e-g	47	247 b-e	45
US-2158	3891 g-l	39	158 e-j	41	1021	5892 c-g	45	240 b-e	46
US-2107	3281 i-l	46	134 g-j	47	1133	5482 d-g	46	223 b-e	47
Cleopatra	4272 f-l	38	165 e-j	40	838	5314 e-g	48	206 c-e	48
Ridge	1722 kl	49	69 ij	49	1368	3218 fg	49	129 de	49
US-1103	349 l	50	15 j	50	2334	1122 g	50	47 e	50
*Mean*	*6112*		*252*		*1029*	*8696*		*359*	
*P-value*	*<0.00001*		*<0.00001*			*<0.00001*		*<0.00001*	

a Mean groups for significant ANOVA within columns were by Tukey test at P<0.05.

b Table is sorted by this column.

Using 640 trees per hectare, the highest fruit production per hectare per year were on the rootstocks US-1688, US-2338, and US-1676, at 11044, 10316, and 9951 kg, respectively. At that same tree density, the same three rootstocks had the highest soluble solids production per hectare, at 440, 414, and 400 kg, respectively. At 640 trees per hectare, solids production per hectare for the rootstocks standard sour orange, Swingle, Cleopatra, and Ridge, had values (ranks) of 343 (9), 274 (21), 165 (40), and 69 kg (49), respectively.

When tree densities were adjusted to the calculated optimum for each rootstock, there were some substantial changes in relative production among the rootstocks. However, two of the same three rootstocks remained at the top of the ranking regardless of whether a standard spacing of 640 trees/hectare or optimized spacing was used, and regardless of whether the calculation was based on fruit production or total soluble solids production. The most productive rootstocks, with each planted at its optimized spacing, were US-2338 [Cm × (Changsha × Pt)], US-1649 (Cm × Sunki), and US-1676 (Cm × Tachibana), with a calculated total soluble solids per hectare per season of 544, 525, and 523 kg, respectively. At the calculated optimized density for trees on standard sour orange, Swingle, Cleopatra, and Ridge, values (ranks) for total soluble solids per hectare were 390 (19), 349 (30), 206 (48), and 129 kg (49), respectively.

### Parentage effects on traits

Eight different parental combinations were represented by two or more hybrids in the trial, with six of the combinations represented by 4-11 hybrids (**Table 7**). Of the 30 rootstock-associated traits evaluated and compared in **Tables 2**–**6**, twenty-six of those were observed with significant effects by parental combination. In general, progeny from the cross Cm × Cleopatra were associated with large tree size, high fruit productivity, large fruit size, a healthy canopy, and high fruit and total solids productivity at 640 trees per hectare. Progeny from Cm × Cleopatra were also generally associated with low total soluble solids concentration, low total acid, and low total solids per MT.

**Table 7:** Rootstock parentage effects on traits.

Trait	Cm × Cleopatra	Cm × Sunki	Cm × Shekwasha	Cm × (Changsha × Pt)	Cm × (Sunki × Pt)	Cm × Tachibana	Sunki × (Cm × Pt)	Shunkokan × Cm	*P-value*
Number of hybrids	5	2	2	4	5	6	7	11	
Tree survival (%)	94	90	100	80	96	93	100	96	*0.13409*
Scion trunk dia^ab^ (mm)	87.7 a	84.6 ab	84.1 ab	74.5 ab	72.9 ab	71.7 ab	68.5 b	66.1 b	*0.00141*
Rootstock trunk dia (mm)	99.3 a	100.5 ab	98.8 ab	92.1 ab	89.2 ab	84.5 ab	91.4 ab	82.6 b	*0.01796*
Scion/Rootstock trunk ratio	0.884 a	0.842 ab	0.849 ab	0.811 ab	0.816 ab	0.846 ab	0.756 b	0.800 ab	*0.01721*
Canopy height (m)	2.04 a	1.95 ab	1.98 ab	1.81 ab	1.76 ab	1.77 ab	1.75 ab	1.70 b	*0.01074*
Canopy dia (m)	2.66 a	2.50 ab	2.45 ab	2.21 ab	2.16 b	2.22 ab	2.10 b	2.06 b	*0.00133*
Canopy volume (m3)	3.70 a	3.13 ab	3.05 ab	2.32 b	2.13 b	2.28 b	2.06 b	1.84 b	*0.00019*
2018 Fruit yield/tree (kg)	5.48	5.55	4.18	5.05	5.00	6.57	5.09	4.26	*0.25004*
2019 Fruit yield/tree (kg)	10.28	9.43	7.88	8.23	7.74	7.60	5.35	5.56	*0.13195*
2020 Fruit yield/tree (kg)	12.68 a	11.41 ab	10.79 ab	11.75 ab	9.20 ab	10.87 ab	6.56 b	7.80 b	*0.00739*
2021 Fruit yield/tree (kg)	15.82 a	14.76 ab	12.63 a-c	10.99 a-c	10.02 bc	12.09 ab	6.71 c	7.24 c	*0.00001*
Fruit yield 2018-21 (kg)	44.26 a	41.15 ab	35.47 ab	36.02 ab	31.97 ab	37.14 ab	23.71 b	24.86 b	*0.00149*
Yield efficiency (kg/m3)	3.86 ab	4.33 ab	3.78 ab	5.24 a	4.53 ab	5.21 a	3.35 b	4.06 ab	*0.00173*
Juice total soluble solids (%)	7.45 c	7.51 bc	7.62 a-c	8.01 a-c	8.05 ab	7.59 bc	8.18 a	7.87 a-c	*0.00027*
Juice acid (%)	0.695 bc	0.696 a-c	0.710 a-c	0.733 a-c	0.781 a	0.687 c	0.763 ab	0.710 a-c	*0.00458*
Juice TSS/acid ratio	10.88	10.99	10.86	11.17	10.51	11.24	10.97	11.32	*0.12530*
Juice percent	53.8	53.6	53.1	54.0	55.0	53.4	54.4	53.9	*0.07597*
Juice color (CN)	36.83 a-c	36.63 c	36.72 a-c	36.87 a-c	36.89 ab	36.78 bc	36.84 a-c	36.93 a	*0.00102*
Weight per fruit (g)	177 a	191 a	174 ab	183 a	173 ab	185 a	161 b	174 a	*0.00013*
Total solids per MT (kg)	40.08 c	40.24 a-c	40.45 a-c	43.33 a-c	44.30 ab	40.53 bc	44.57 a	42.47 a-c	*0.00110*
2018-19 Canopy health	3.64 a	3.32 ab	3.64 ab	3.22 ab	3.33 ab	3.23 ab	3.11 b	3.18 ab	*0.0296*
2020-21 Canopy health	4.24 a	3.73 ab	4.03 ab	3.28 b	3.38 b	3.39 b	3.35 b	3.34 b	*0.00010*
2021 Canopy color	3.76 a	3.30 a-c	3.57 ab	3.21 bc	2.97 c	3.03 bc	3.28 bc	3.13 bc	*0.00002*
2021 Canopy thickness	3.91 a	3.20 ab	3.60 ab	3.06 b	2.90 b	2.94 b	3.09 b	3.01 b	*0.00003*
2021 HLB Symptoms	2.40 c	2.74 a-c	2.59 a-c	2.76 a-c	3.03 ab	3.11 a	2.65 bc	2.73 a-c	*0.00159*
2021 Premature drop (%)	32.5 c	39.4 a-c	43.3 a-c	43.0 a-c	40.1 bc	41.9 a-c	51.7 ab	55.2 a	*0.00039*
Combined health rank	6.3 b	20.0 ab	13.1 ab	27.8 a	30.1 a	30.2 a	28.9 a	30.5 a	*0.00076*
Fruit/ha at 640 trees (kg)	9119 a	8373 a-c	7494 a-c	7277 a-c	6151 a-c	7349 ab	4247 c	4812 bc	*0.00013*
Solids/ha at 640 trees (kg)	359 a	331 ab	298 ab	308 ab	266 ab	293 ab	186 b	201 b	*0.00127*
Optimized trees per hectare	753 b	828 ab	859 ab	1015 ab	1046 ab	1008 ab	1104 a	1105 a	*0.01932*
Fruit/ha optimized (kg)	10299 ab	10584 a-c	9447 a-c	10794 ab	9291 a-c	10789 a	6693 c	7912 bc	*0.00033*
Solids/ha optimized (kg)	404 ab	420 ab	376 ab	461 a	401 ab	429 a	294 b	330 ab	*0.00539*

a Mean groups for significant ANOVA within rows were by Tukey test at P<0.05.

b Table is sorted by this row. This comparison does not include parental combinations represented by only one clone. Shading highlights significant parental combinations for each trait, with blue shading indicating significant positive effect and yellow indicating significant negative effect.

In contrast, progeny from the cross Sunki × (Cm × Pt) generally induced small tree size, low fruit productivity, small fruit size, a less healthy canopy, and low fruit and solids productivity per hectare. However, progeny from the cross Sunki × (Cm × Pt) generally induced high total soluble solids concentration, high total acid, and high total solids per MT.

Among the other six progeny groups, only the cross Cm × Shekwasha did not show a significant positive or negative influence on some traits. Progeny from the cross Cm × Sunki generally were associated with high fruit weight and low juice color. Progeny from the crosses Cm × (Changsha × Pt) and Cm × Tachibana generally were associated with high yield efficiency, high fruit weight, and high fruit and solids productivity per hectare at optimized spacing, while combined with small canopy volume and low canopy health scores.

Progeny from the cross Cm × (Sunki × Pt) induced high juice total acids, soluble solids, and color, but combined with small tree size and low canopy health. Progeny from the cross Shunkokan × Cm induced high juice color and fruit weight, but it was combined with small tree size, low fruit yield, low tree health ratings, and low yield per hectare.

### Heritability

Estimates of additive genetic variance were obtained by partitioning the genetic components of each trait using two methods, with similar results (**Table 8**). Pedigree-based estimates of narrow-sense heritability (*h* ^2^ ) ranged from 0.04 to 0.73, with a value of *h* ^2^ >0.25 for 20 of the 31 measured traits. Traits associated with tree size, including both canopy size and trunk diameter tended to have high heritability (*h* ^2^ =0.49-0.73), while those associated with fruit quality were lower (*h* ^2^ =0.04-0.33). Traits measured in later years exhibited higher heritability than those measured on younger trees. For example, *h* ^2^ of fruit yield steadily increased from 0.1 in 2018 to 0.42 in 2021. These data indicate that there is a strong genetic component underlying rootstock-mediated influence on traits measured on the common sweet orange scion, and the extent of additive genetic variation underlying trait variation in this set of hybrids highlights the potential of selective rootstock breeding to meet modern challenges in citrus cultivation, including tolerance to HLB. In fact, cumulative yield (2018-2021), and canopy health and thickness measured in 2021 were highly heritable (*h* ^2^ >0.46), indicating that metrics for HLB tolerance will be responsive to selection in future rootstock breeding cycles.

**Table 8:** Heritability (h^2^) of rootstock traits based on phenotypic performance in the field trial.

Trait	h^2^ (rrBLUP)	h^2^ (MCMCglmm)
Tree survival and size		
Tree survival (%)	0.17	0.17 (0.06-0.28)
Scion trunk dia (mm)	0.73	0.73 (0.63-0.82)
Rootstock trunk dia (mm)	0.65	0.65 (0.54-0.77)
Scion/Rootstock trunk ratio	0.68	0.68 (0.57-0.78)
Canopy height (m)	0.49	0.48 (0.35-0.61)
Canopy dia (m)	0.66	0.66 (0.55-0.77)
Canopy volume (m3)	0.66	0.64 (0.53-0.76)
Fruit crop		
2018 Fruit yield/tree (kg)	0.10	0.08 (0-0.17)
2019 Fruit yield/tree (kg)	0.34	0.34 (0.20-0.48)
2020 Fruit yield/tree (kg)	0.27	0.24 (0.12-0.37)
2021 Fruit yield/tree (kg)	0.42	0.40 (0.27-0.54)
Fruit yield 2018-21 (kg)	0.46	0.45 (0.31-0.59)
Yield efficiency (kg/m3)	0.20	0.19 (0.08-0.31)
Fruit quality		
Juice total soluble solids (%)	0.31	0.31 (0.19-0.45)
Juice acid (%)	0.27	0.29 (0.16-0.42)
Juice TSS/acid ratio	0.05	0.04 (0-0.1)
Juice percent	0.17	0.16 (0.05-0.28)
Juice color (CN)	0.04	0.05 (0-0.1)
Weight per fruit (g)	0.24	0.37 (0.24-0.49)
Total solids per MT (kg)	0.33	0.34 (0.21-0.47)
Tree health and HLB symptoms		
2018-19 Canopy health	0.21	0.20 (0.09-0.32)
2020-21 Canopy health	0.58	0.58 (0.46-0.70)
2021 Canopy color	0.39	0.39 (0.25-0.52)
2021 Canopy thickness	0.48	0.49 (0.36-0.62)
2021 HLB Symptoms	0.27	0.27 (0.15-0.40)
2021 Premature drop (%)	0.22	0.22 (0.11-0.35)
Combined health rank	—	—
Production per area and optimum		
Fruit/ha at 640 trees (kg)	0.41	0.40 (0.27-0.54)
Solids/ha at 640 trees (kg)	0.37	0.34 (0.21-0.48)
Optimized trees per hectare	0.66	0.63 (0.51-0.75)
Fruit/ha optimized (kg)	0.22	0.21 (0.10-0.33)
Solids/ha optimized (kg)	0.19	0.17 (0.06-0.28)

**Figure 2:**
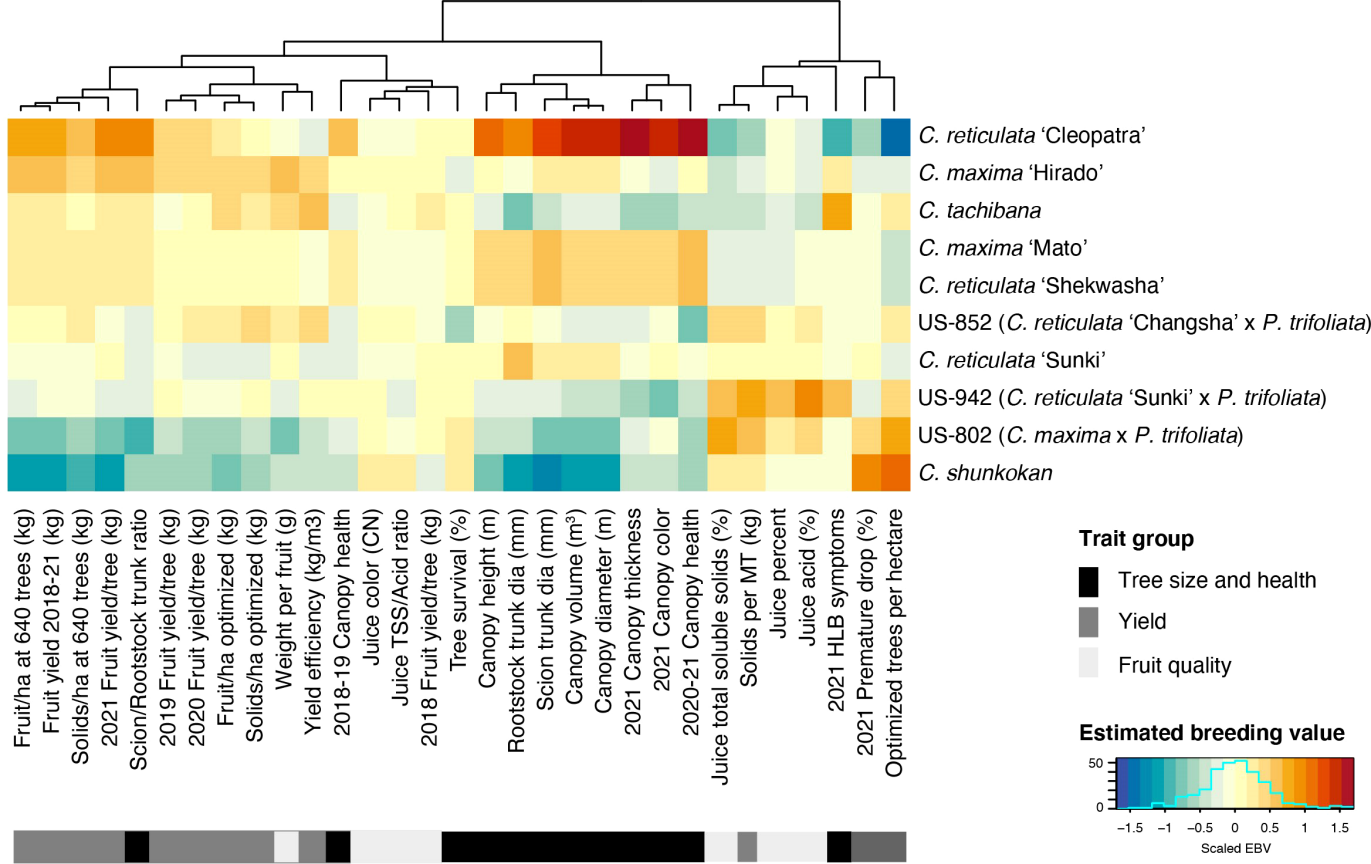
Estimated breeding value of parents. REML linear mixed models for each trait were used to predict the EBV of each parent. Each row represents a cultivar with the cultivar name listed on the y-axis. Cultivars were ordered from highest to lowest fruit yield (2018-2021). Trait names are shown on the x-axis and traits were grouped into three broad categories: tree size and health, yield, and fruit quality. The estimated breeding values for each trait/cultivar combination are relative to the intercept of each model with positive values represented by warmer orange and red colors and negative values by cooler blue colors.

**Figure 3:**
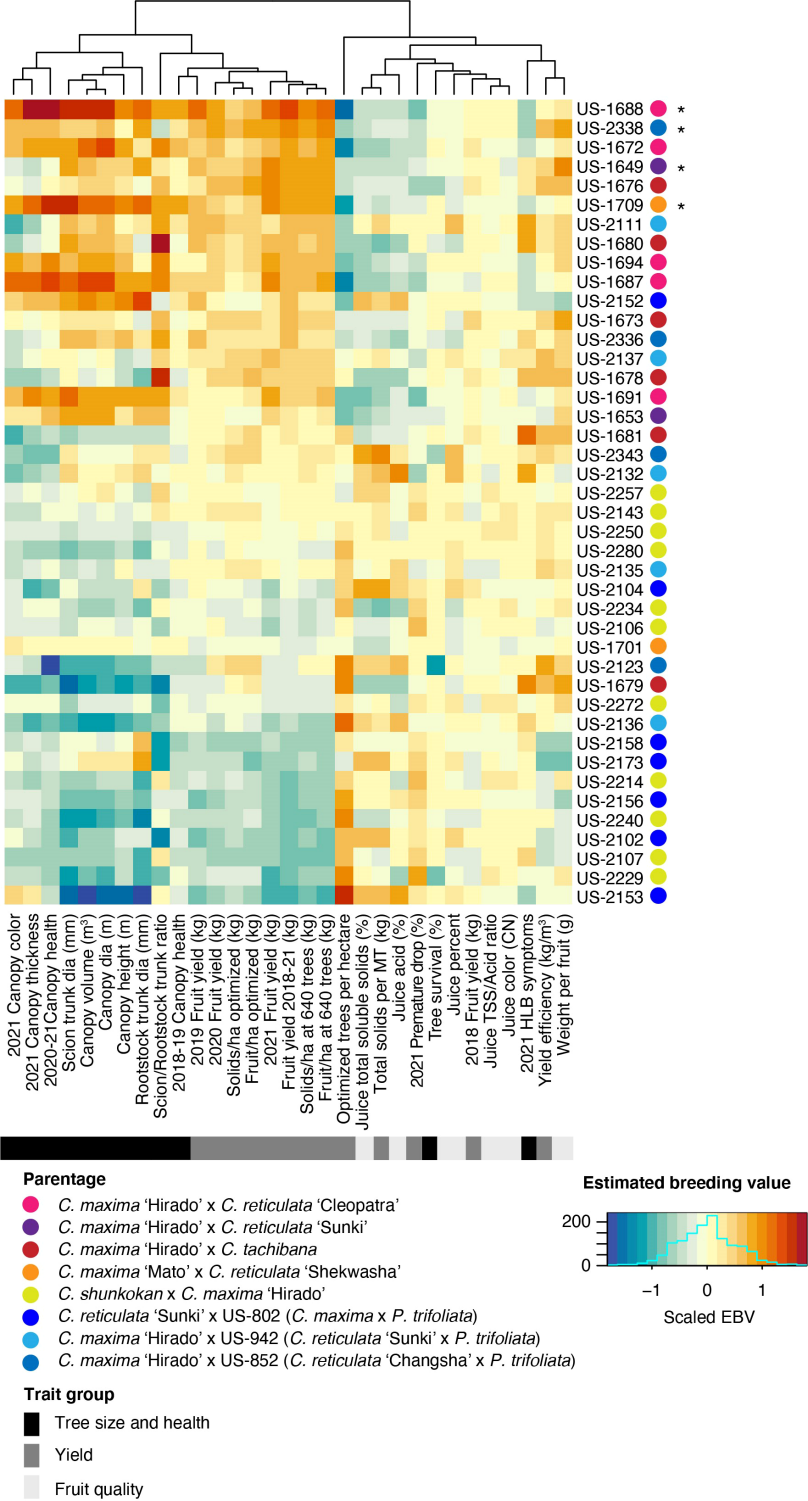
Estimated breeding value of rootstock hybrids. REML linear mixed models for each trait were used to predict the EBV of each hybrid. Each row represents a hybrid with the name listed on the y-axis. Hybrids were ordered from highest to lowest fruit yield (2018-2021), with higher yielding hybrids on the upper portion of the heatmap. The parentage of each hybrid is indicated in the key. Trait names are shown on the x-axis and traits were grouped into three broad categories: tree size and health, yield, and fruit quality. The estimated breeding values for each trait/hybrid combination are relative to the intercept of each model with positive values represented by warmer orange and red colors and negative values by cooler blue colors.

Estimated breeding values (EBV) for each trait were predicted for the 42 rootstock hybrids in families with more than two members, as well as their parents. Comparison of breeding values across parents and traits reiterates the trends from line averages. Cleopatra contributes alleles that are associated with large tree size and increased fruit yield, but reduced fruit quality characteristics (**Figure 2**). Parental cultivars with trifoliate ancestry were associated with reduced HLB disease symptom scores, while these parents gave rise to smaller trees and lower yield, but elevated fruit quality. EBVs for the hybrids showed a similar relationship between traits with higher yielding rootstocks typically having larger sizes, but decreased quality characteristics (**Figure 3**). When ranked by the predicted breeding value for cumulative fruit yield from 2018-2021, hybrids derived from Cm × Cleopatra, Cm × Sunki, and Cm × Tachibana tended to outperform hybrids with trifoliate ancestry, although there were exceptions. Pedigree-based prediction of EBVs in this trial of rootstock hybrids revealed that there is substantial additive genetic variation for rootstock-mediated influence on traits associated with tree performance in HLB endemic conditions.

## Discussion

This study of the Picos 2014 rootstock trial revealed significant rootstock modulation of a broad array of traits of horticultural significance, including tree size, fruit yield, fruit quality, tree canopy health and HLB symptoms, in a Valencia sweet orange trial containing 50 rootstocks and 100% infected by CLas. Although we were not able to identify the relative influence of CLas on each trait, it was clear that CLas had a strong effect on many, if not all traits in this trial. In a previous report, a comparison of Valencia sweet orange tree growth on Swingle rootstock in Florida without and with CLas infection, indicated a 19% reduction in scion trunk cross sectional area (TCSA) at seven years of age as a result of CLas infection (Bowman et al., 2016a). A similar estimate can be made for the effect of CLas in this Picos 2014 trial because of data from a previous rootstock trial with Valencia scion planted in 1999 on the exact same site and containing Swingle and standard sour orange rootstocks. In this previous trial (Picos 1999) on the same site as the Picos 2014 trial, Valencia trees on Swingle and standard sour orange rootstocks had scion TCSA of 7287 and 9502 mm^2^, respectively, at seven years after planting and prior to CLas infection at the site. Converting scion trunk diameter (from **Table 2**) for the Picos 2014 trial, indicates TCSA for Valencia trees on Swingle and standard sour orange rootstocks of 4754 and 7103 mm^2^, respectively, at seven years of age. Since the trees in the Picos 2014 trial are 100% infected with CLas, this comparison indicates a reduction in Valencia tree TCSA growth resulting from CLas infection of 35% and 25%, on Swingle and standard sour orange rootstocks, respectively. We propose that the estimation of a larger reduction in TCSA from CLas infection in this present study, as compared with the previous report (Bowman et al., 2016a), may be a result of the trees becoming infected at an earlier age in the Picos 2014 trial. The previous published study (Bowman et al., 2016a), was planted in 2008 during the early stages of CLas spread through Florida, while the current study was planted in 2014 and after CLas was completely ubiquitous in the area of the trial. It can be noted that although all trees were infected and there was a clear reduction in growth from CLas infection in this Picos 2014 trial, through seven years of age there was no tree death on Swingle, standard sour orange, or Cleopatra rootstocks. The effect from CLas infection with most rootstocks was primarily to weaken trees, not to cause tree death.

The two highly-popular rootstocks included in the trial, standard sour orange and Swingle, exhibited excellent tree survival and were intermediate in overall tree performance. The other two standard commercially-available rootstocks Cleopatra and Ridge were among the overall poorest performers in this trial. Rootstock effect on tree size was divided into five categories, based on scion trunk diameter (**Table 2**), with standard sour orange and Cleopatra producing among the largest trees in the trial, and Swingle producing medium-large trees. Ridge exhibited poor tree survival (50%) and a small size for the surviving trees. Some trees on Ridge rootstock that died showed trunk rot near the soil surface, and we postulate that Ridge suffered substantially in the trial from Phytophthora root and foot rot, as has previously been observed for *C. sinensis* when used as a rootstock (Albrecht and Bowman, 2004).

When sweet orange is grown for juice production, total soluble solids of the juice is of critical importance to commercial success. Although standard sour orange is generally considered to provide good fruit quality for grafted scions, under the conditions of this trial it produced the lowest juice total soluble solids concentration and one of the lowest values for TSS per metric ton of fruit. However, trees on standard sour orange also produced the lowest juice total acids, resulting in among the highest TSS:acid ratios, perhaps justifying the perception of inducing high fruit sweetness, even if the actual fruit TSS values are low. While the perception of sweetness is an important consideration in citrus fruit grown for the fresh market, the actual measured amount of total soluble solids is the factor of critical importance for sweet oranges grown for juice.

**Figure 4:**
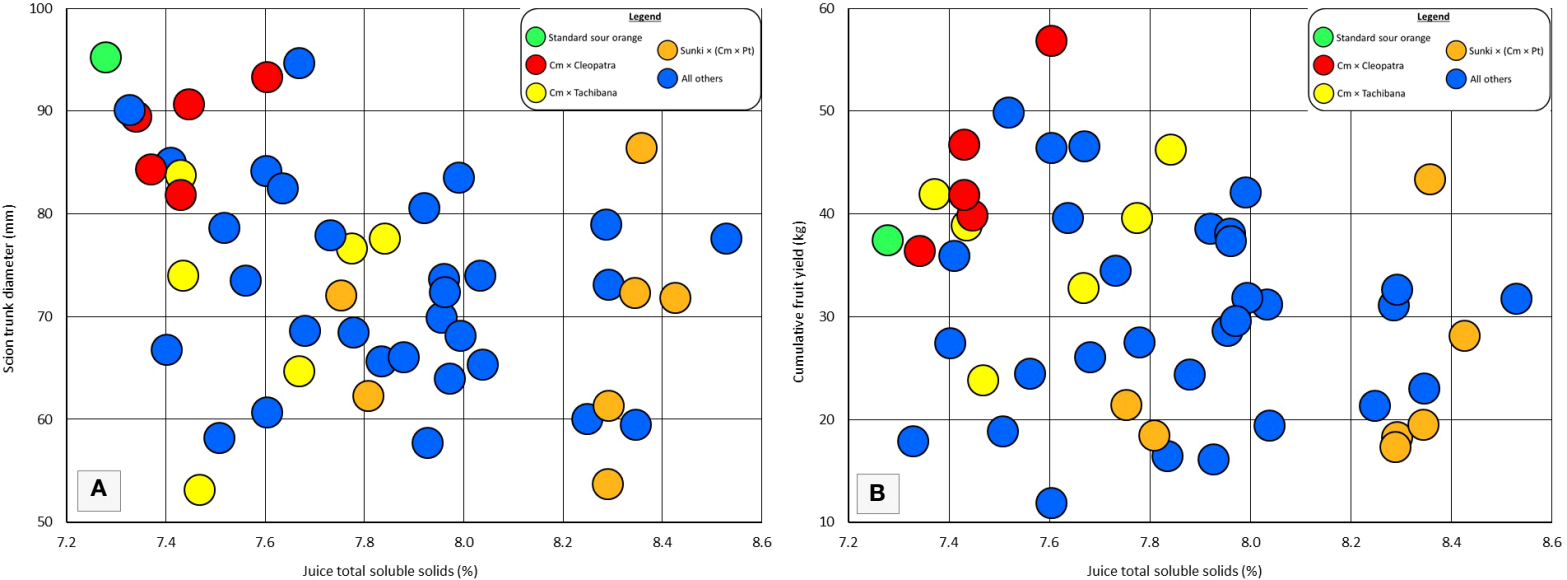
Individual and progeny rootstock effects for juice total soluble solids in comparison with **(A)** scion trunk diameter, and **(B)** cumulative fruit yield. All Other = rootstocks that are not standard sour orange or the three hybrid progeny groups specifically identified.

Rootstock influence on tree vigor may be associated with the total soluble solids of fruit. Rough lemon, Volkamer lemon, and US-802 are rootstocks that induce high vigor in the grafted scion, and have been observed to induce relatively low TSS in the fruit of grafted sweet orange trees (Bowman and Joubert, 2020). Among the rootstocks in this trial, the six rootstocks that induced the largest tree size (including standard sour orange), also produced among the lowest fruit TSS (**Figure 4A**), suggesting that very high rootstock vigor may be associated with low TSS. However, among the remaining 44 rootstocks, there was not a strong association between rootstock influence on tree size and rootstock influence on fruit TSS. Within each category of rootstocks for fruit TSS: low TSS (7.3-7.8%), medium TSS (7.8-8.1%), and high TSS (8.2-8.6%), there were some rootstocks that induced relatively low vigor, medium vigor, and high vigor. The strong clustering of hybrids for vigor and fruit TSS within some parental combinations, such as Cm × Cleopatra, does suggest that these two traits may be associated within some progeny groups.

Similarly to vigor, rootstock influence on fruit productivity may be associated with TSS of fruit (**Figure 4B**). Among the rootstocks in this trial, the five with highest cumulative yield per tree were in the low TSS category (7.3-7.8%). But this association was not very strong for the remaining 45 rootstocks, and there was one hybrid which combined good cumulative yield with high fruit TSS. Again, clear clustering of hybrids for cumulative yield and fruit TSS within some parental combinations may indicate linkage of these two traits within some progeny groups.

Among the 50 rootstocks included in this trial, there was a broad diversity in the influence on traits of the grafted trees in the field. In addition to clear specific rootstock differences, the comparison between the eight parental combinations represented by more than one hybrid indicated than most parental combinations exhibited a tendency to produce hybrids with a specific combination of characteristics of horticultural importance (**Table 7**). There were outliers within each progeny group, and no progeny group tended to have the favored trait in every category. However, progeny from the cross Cm × Cleopatra generally were among the best for the important traits, while progeny from the cross Sunki × (Cm × Pt) were among the worst. This trend was supported by the estimated breeding value predictions across traits (**Figures 2**, **3**). While field testing individual rootstocks will be required to identify the specific hybrids most likely to provide outstanding field performance, the results suggest that choosing the optimum parental combinations to be included in field trials can substantially increase the opportunity to discover those most superior hybrids. Additionally, estimates of heritability indicate that this population of hybrids will be responsive to selection for increased yield and tree and canopy size and health metrics. The goal of pedigree-based estimates of heritability is to partition additive genetic variance from other sources of variation, but there are conflicting reports on the extent that these methods may overestimate narrow-sense heritability (Joshi et al., 2020). One potential issue is that maternal effects cannot be separated from additive effects. In this study, Cm is commonly used as the seed parent, but a few hybrids were generated with Cm as a pollen parent. Breeding values for Cm as the maternal and paternal parent were similar, but future work to disentangle the effects of maternal source and pedigree using genetic information will resolve this issue. An additional benefit of incorporating genetic information to partition genetic components of rootstock-mediated traits is that genome-wide information will capture shared ancestry not reflected in the pedigree. While the integration of genetic information (either based on pedigree or genetic variation) with phenotypic data using mixed models is a common practice for 1) dissecting genetic components of quantitative traits and 2) predicting additive genetic values (EBVs), including for tree crop species (Martínez-García et al., 2017; Fernández-Paz et al., 2021; Cañas-Gutiérrez et al., 2022), this is an early application for dissecting genetic control of rootstock-mediated effects. Such studies in citrus have focused on scion-related traits, including implementation of genomic selection for fruit quality (Gois et al., 2016; Imai et al., 2016; Minamikawa et al., 2017; Imai et al., 2019). Examination of the genetic control of rootstock traits in citrus has, so far, been limited to estimates of broad-sense heritability in biparental populations (Siviero et al., 2006; Lima et al., 2018), although similar approaches have been applied in rootstock studies in a few other species (Reyes-Herrera et al., 2020; Fernández-Paz et al., 2021). This is the first study to estimate narrow-sense heritability and calculate EBVs for a broad range of rootstock modulated traits in citrus.

This trial is a part of the overall SuperSour strategy (Bowman et al., 2021) to collect field performance data on a large group of diverse rootstock hybrids that will be used to: 1) identify superior new rootstocks, and 2) identify the most promising parental combinations, and 3) map many important rootstock traits to allow pre-selection in the next generation of rootstock breeding. Prior work to map traits for citrus rootstock breeding have generally been of limited scope in target traits and focused on a relatively small range of parental combinations (Xiang et al., 2010; Curtolo et al., 2018; Huang et al., 2018; Lima et al., 2018; Shimizu et al., 2020; Soratto et al., 2020). In contrast, the SuperSour strategy incorporates a rather large diversity of parental material and includes assessment for a broad range of important rootstock traits, and consequently is aimed to have broad applicability. Although the specific Picos 2014 trial contains only 45 of the 350 hybrids in the mapping population, the observed significant rootstock effects on a broad range of measured traits, high heritability (h^2^) of many important traits, and strong association of particular parental combinations with particular trait combinations suggest that the strategy will be effective. Ranking indices to use for systematic selection of superior citrus rootstocks in trials have been described (Costa et al., 2020a), and provide a coherent methodology for selection, although that approach does not significantly contribute to mapping of important traits and pre-selection of superior new hybrid rootstocks.

Several new hybrid rootstocks included in this trial exhibited superior fruit production, juice quality, fruit and juice yield efficiency, and tree health in comparison to the standard rootstocks. Among the four standard rootstocks in the trial, standard sour orange appeared overall the best despite very poor juice total soluble solids values. Swingle rootstock exhibited better fruit quality than standard sour orange in the trial, but still trailed it in overall productivity even when taking total soluble solids and tree size into account. In comparison to standard sour orange, six and eight of the new rootstocks exhibited higher values for annual fruit yield per hectare or total solids per hectare, respectively, at a standard spacing of 640 trees per hectare (**Table 6**). The similar comparisons with Swingle rootstock indicated higher values for 16 and 19 of the new hybrid rootstocks, respectively. Statistical comparisons among the rootstocks indicated significant rootstock differences for all traits examined, except TSS/acid ratio. The best performing new hybrid rootstocks were significantly better than many rootstocks in the trial for critical traits such as fruit yield, yield efficiency, soluble solids per metric ton, and productivity per hectare. However, standard sour orange exhibited overall good performance, and these best new hybrid rootstocks were not significantly better than standard sour orange in any of the key traits except soluble solids per metric ton.

Tree size within the trial varied from the largest like those on standard sour orange, to those on US-1103, which made trees only about 12% the canopy volume of trees on standard sour orange (**Table 2**). With this large differential in rootstock effect on tree size, it was not surprising that comparisons among rootstocks which took into account yield efficiency or planting at a tree density adjusted by tree size provided somewhat different relative rankings of the rootstocks for fruit productivity (**Table 6**). Adjusting planting density for anticipated tree size at 7 years, estimates of productivity for each rootstock indicated that 14 of the hybrid rootstocks would have superior fruit yield per hectare compared to standard sour orange, while 18 of the hybrid rootstocks would have superior total soluble solids yield.

One purpose for the trial was to identify the superior new hybrid rootstocks that may be used for commercial production of sweet orange under HLB-endemic conditions. For this assessment, relative fruit or total solids productivity per season are probably among the most important criteria to be considered (**Table 6**). When planted at the tree density in the trial, the rootstocks US-1688, US-2338, US-1676, and US-1709 were ranked as the highest producers per hectare per season for either amount of fruit or total soluble solids. When calculated planting density is varied to optimize for tree size, the rootstocks US-2338, US-1676, US-1649, and US-1688 or US-2123 (depending on whether using fruit or total solids) were ranked as the highest producers per hectare per season. Despite good productivity, the rootstocks US-1676 and US-2123 were observed to have reduced tree survival through the first seven years, and were therefore eliminated from among the top performers in the trial. Consequently, US-1649, US-1688, US-1709, and US-2338 were considered the four most promising new rootstocks in this trial. Release of these rootstocks for commercial use is being considered, pending the evaluation of continuing performance in this trial and the results from other trials. It is important to note that the performance of the rootstocks described in this study were evaluated at only one location, and with environmental and management conditions as indicated. Performance of these rootstocks may be significantly different in other citrus growing regions or with different management. As is the case with all very new rootstocks, additional testing should be conducted to validate observations of superior performance.

Despite the observations that particular parental combinations appeared to be generally superior to others in likelihood of yielding superior hybrid rootstocks, it can be noted that the four most promising rootstocks identified above are the product of four different parental combinations, and most parental combinations produced some favorable rootstock traits among progeny. While the evaluation of performance for specific parental combinations and calculation of breeding value for parents can be valuable tools to guide the next generation of rootstock breeding, it appears that many different parental combinations possess the potential to yield superior new hybrids. In addition, high genetic diversity among rootstock breeding materials, rather than focus on a few specific genetic combinations proven good in the past, provides the best opportunity to identify novel superior types along with good insurance against unanticipated future biotic and abiotic challenges to citrus production.

It should be noted that overall fruit productivity in the trial would be considered poor in comparison with pre-HLB Valencia sweet orange production in Florida. Although rootstock was a powerful influencer of tree productivity and fruit quality in the trial, even the best rootstocks were unable to completely overcome the debilitating effects from HLB disease: Fruit production per tree or per hectare was still relatively low. It is likely that the most profitable citrus production in the HLB-endemic environment will require the combination of best scion and best rootstock, along with improved management practices that lessen disease impact and improve overall tree health.

## Conclusions

This study revealed significant citrus rootstock modulation of a broad array of traits of horticultural significance, including tree size, fruit yield, fruit quality, tree canopy health and HLB symptoms, in a sweet orange trial containing 50 rootstocks and strongly affected by HLB. Comparison of eight different parental combinations among the hybrids identified significant differences in parental influence on progeny traits that can guide future breeding. There is substantial additive genetic variation underlying these traits, indicating that this population will be responsive to selection for exceptional tree performance in HLB-endemic environments. Several new hybrid rootstocks, including US-1649, US-1688, US-1709, and US-2338, exhibited superior performance and were identified for additional testing and potential commercial release.

## Data availability statement

The raw data supporting the conclusions of this article will be made available by the authors, without undue reservation.

## Author contributions

KB carried out conceptualization, methodology, investigation, validation, supervision, project administration, funding acquisition, data curation, formal analysis, and visualization, as well as writing, reviewing, and editing the draft. KB created the hybrids included in the study. GM made significant contributions to fruit quality evaluation, as well as reviewing and editing the draft. DS calculated the variance components, trait heritability, and estimated breeding values, and wrote those associated sections of the manuscript, as well as reviewing and editing the draft. All authors contributed to the article and approved the submitted version.
